# Cognitive fitness and mental health outcomes following COVID-19 lockdowns in Australia

**DOI:** 10.1038/s41598-025-29205-w

**Published:** 2025-12-25

**Authors:** Sabina Kleitman, Dayna J. Fullerton, Lisa M. Zhang, Madeleine T. King, Eugene Aidman

**Affiliations:** 1 School of Psychology, Faculty of Science, The University of Sydney, Sydney, NSW Australia; 2 Divison of Human and Decision Sciences, Defence Science and Technology Group, Edinburgh, SA Australia; 3 School of Biomedical Sciences and Pharmacy, University of Newcastle, Callaghan, NSW Australia

**Keywords:** Human behaviour, Public health

## Abstract

The uncertainty, stress and prolonged social isolation caused by pandemics significantly affect mental health. This paper presents two cross-sectional studies examining key psychological variables associated with mental well-being, distress, and anxiety following COVID-19 lockdowns in Australia, utilizing the Cognitive Fitness Framework (CF2). We also report the development and validation of the COVID-19 Character Growth Awareness (CGA) scale. In Study 1 (N = 417), impulsivity/lack of self-control, resilience/adaptability and COVID-19 CGA predicted 6% of variance in post-lockdown mental well-being beyond all other variables. From the same block of variables, only CGA predicted a significant amount of variance (3%) in mental well-being recovery. In Study 2 (N = 1898), intolerance of uncertainty correlated positively with psychological distress and anxiety, while CGA correlated negatively while controlling for other covariates. These results highlight the importance of psychological strengths and vulnerabilities in enabling adaptation and recovery following public health crises. The COVID-19 CGA emerged as one of those strengths. The results also help unify the theory of resilience as a trait, process, and outcome. Understanding what assists the maintenance and recovery of mental health amid the challenges and isolation of pandemics can help develop strategies to mitigate the impacts of future crises that present similar challenges.

**Supplementary Information:**

The online version contains supplementary material available at 10.1038/s41598-025-29205-w.

## Introduction

The uncertainty, stress, and prolonged social isolation caused by major public crises, such as pandemic-related lockdowns, can significantly affect mental health^1,2^. It is important to understand the psychological factors that influence how people adapt to crises. Identifying these factors should help researchers, policymakers, and other stakeholders understand how to foster resilience, promote psychological recovery in affected populations, and prepare for future challenges. These insights also guide long-term strategies to support post-traumatic growth (positive change following adversity or trauma^3^) and societal well-being, enabling individuals and communities to emerge stronger from challenging events.

Adaptation can affect critical choices (e.g., compliance with public health orders, see^4,5^) and mental health during a crisis^2,6^. The emerging Cognitive Fitness Framework (CF2) and the transdisciplinary expert consensus that followed^7^, offer a valuable model in this context because it proposes the key components of such adaptation^7,8^. A critical phase within this framework is the recovery stage, which allows the mind to reset and strengthen, forming a foundation for the next challenge^8^. During this stage, the person reflects on the difficulties they have endured and engages in reparative activities (e.g., sleep, proper nutrition, social connection). In operational and high-stakes contexts, the recovery phase is critical to prevent cognitive fatigue and burnout. The emergency stages of the COVID-19 pandemic, such as lockdowns and post-lockdowns, presented a unique opportunity to examine the psychological constructs proposed by the CF2 model which help us manage and recover from difficult and uncertain times within an ecologically valid context. There are lessons to be learned about what facilitates positive adaptation during public crises from the challenges inflicted by the COVID-19 pandemic of 2020–2023.

Cognitive Fitness is a multi-faceted and differentially malleable capacity to deliberately deploy neurocognitive resources, knowledge, and skills to meet challenging demands and sustain performance^8^. The overarching goal of this paper was to validate key constructs outlined by the CF2^7,8^. In this paper, we focus on the constructs of impulsivity, self-control, resilience, adaptability, intolerance of uncertainty and a newly developed measure of COVID-19 Character Growth Awareness (CGA), and their association with the maintenance and recovery of mental health following COVID-19 pandemic lockdowns. Each construct, its relationships with other constructs, and proposed relationships with mental functioning are discussed below.

This paper presents two cross-sectional studies examining key psychological variables associated with mental health during and after two COVID-19 lockdowns in Australia. We start by outlining the psychological constructs of interest and their respective measurement tools: the CF2 constructs (resilience, impulsivity, self-control and adaptability in Study 1), intolerance of uncertainty (Study 2), and pandemic-related character growth awareness (examined in both studies). We then outline the two studies, including their respective outcome measures (mental health metrics), explanatory variables (CF2 constructs and other covariates), and hypotheses. The results are then presented, and the findings are discussed in the context of existing literature. The methods (including statistical analysis) are presented after the Conclusions.

## Psychological constructs and measurement tools

### Cognitive fitness framework (CF2) constructs

#### Impulsivity and self-control

Executive functions, namely inhibitory control (ability to control automatic, goal-irrelevant or incorrect responses to adhere to a rule or to achieve a goal^9^), form the foundational phase of the CF2^8^. To examine the role of these attributes, we included measures of impulsivity, defined as a tendency toward rapid unplanned reactions to stimuli without regard for adverse consequences and self-control, the capacity to regulate attention, emotions and behaviour to achieve some long-term goals. Specifically, we employed the UPPS-P Scale to capture five impulsive traits: the tendencies to act rashly under extreme negative or positive emotions, to act without thinking, to seek out novel and thrilling experiences, and the inability to remain focused^10^. Self-control was measured using the Brief Self-Control Scale^11^. Given conceptual overlap and previously reported strong negative relationships between self-control and impulsivity^12^, we expect the measures to converge into a composite.

#### Resilience and adaptability

While several conceptualisations exist, resilience typically refers to positive adaptation in the face of stressors^13^, and it can assist the deployment of neurocognitive resources to sustain performance. One approach conceptualises resilience as a psychological trait or ability (see^14^ for a review), whilst most recent research conceptualises resilience as a process whereby resources protect against the negative impact of stressors to produce positive outcomes^15^. The American Psychological Association (APA)^16^ defines resilience as “the process and outcome of successfully adapting to difficult or challenging life experiences, especially through mental, emotional, and behavioural flexibility and adjustment to external and internal demands” (APA, “Resilience”). Consistent with previous research^15^, we adopt an integrative approach that resilience involves all three aspects—stable psychological resources, a dynamic process of utilising resources and responding to stressors, and an adaptive outcome. Related to resilience, adaptability is the capacity to adjust behaviours, thoughts, and emotions in response to changing or uncertain circumstances^17,18^. These constructs are relevant in the context of the pandemic as the situation constantly changed, produced new challenges, and required the population to adjust their behaviour and lifestyle to meet demands. Previous research has supported the roles of resilience and adaptability in mental health during the COVID-19 pandemic^19^.

We used well-validated measures of the constructs—the Connor-Davidson Resilience Scale (CD-RISC^20^) to measure resilience and the Individual Adaptability Scale (I-ADAPT^18^) to measure adaptability. Theory and previous research have indicated that these measures share strong conceptual overlap and strong positive correlations, with metrics from both measures converging when factorised^15,21^. Both measures were combined in a composite to mitigate collinearity problems.

One of the most recent schools of thought views resilience as a complex interaction of various socio-ecological factors: “the process of biological, psychological, social, and ecological systems interacting in ways that help individuals to regain, sustain, or improve their mental wellbeing” in the face of risk factors^22^, p. 441). To address this view, we captured and controlled for the most salient aspects of the ecological system, including social support and a comprehensive selection of situational and demographic characteristics.

### COVID-19 character growth awareness (COVID-19 CGA)

There is a need to consider dynamic, context-specific aspects of the positive adaptation process^23,24^. In the context of COVID-19, we propose that reflection and awareness of how the pandemic’s challenges built one’s strength, resilience, and understanding of how to adapt to changing rules and restrictions were associated with positive outcomes such as recovery and maintaining psychological health. This view was informed by theories of posttraumatic growth (PTG), resilience, and metacognition.

PTG is defined as “positive psychological changes experienced as a result of the struggle with traumatic or highly challenging life circumstances”^3^, p. 3). The Posttraumatic Growth Inventory (PTGI) captures five domains: personal strength, relating to others, new possibilities, appreciation of life, and spiritual and existential change^25^. A Spanish study conducted during the pandemic found that scores on the PTG *personal strength* dimension increased following the end of strict COVID-19 lockdown regulations, and this was the only PTG dimension that showed an overall increase over the 4 months^26^. Accordingly, the proposed COVID-19 CGA construct aligns with the domain of personal strength defined by experiencing an increased sense of self-reliance, strength, and confidence, and perceiving oneself as a survivor rather than a victim^3^.

The experience of the COVID-19 pandemic was associated with increased strength and character because of enduring prolonged periods of uncertainty, change, and adversity. As Vazquez et al.^27^ noted, evaluating these perceptions of growth *whilst the pandemic was still ongoing* captured a response or strategy for coping with the pandemic. This differentiates it from the broad PTG construct, which is conceptualised as a *long-term*, deep transformation occurring *after* a traumatic event and encompassing several additional and broader changes (see^3^). Another key conceptual difference is that PTG theory defines trauma as an objectively or subjectively highly stressful and life-altering event. In examining the CF2 model^8^, we were interested in performance under demanding, challenging conditions, particularly high-risk occupations. In the context of the COVID-19 pandemic, the experience of trauma was highly variable among individuals, as the pandemic elicited different degrees of disruption to daily life (which we captured and controlled for statistically, see also^2,4^), though it was not necessarily a life-altering event for all.

Thus, we developed the COVID-19 CGA Scale to capture awareness of growth through the COVID-19 pandemic and examine its associations with different mental health metrics.

#### COVID-19 CGA scale development: theory and items

Capturing metacognitive reflections on resilience and character growth is crucial for understanding post-traumatic growth because metacognition—thinking about one’s own thinking—allows individuals to evaluate, process, and integrate their trauma-related experiences. Most theories of metacognition focus on metacognitive knowledge (beliefs and knowledge about one’s cognitive abilities and strategies; see^28^ for a review). We used Efklides’^28^ theory of metacognition as it provides an understanding of how individuals monitor, control, and evaluate their cognitive processes via emotional lenses. In doing so, the theory differentiates between metacognitive knowledge and metacognitive experiences (which include feelings and judgments). In the context of PTG, Efklides’ concept of affective metacognitive experiences can help our understanding of how individuals draw on self-beliefs and knowledge about their affective selves, their adaptive coping, and their character growth. This reflection is essential for making meaning from traumatic experiences, a key aspect of PTG. By understanding their own beliefs about their abilities to cope, resilience and strategies, individuals can consciously reframe their trauma and foster a sense of growth.

The items’ development was then guided by the theories of resilience (see^15^ for a review) and posttraumatic growth^25^, and relevant scales—Connor-Davidson Resilience Scale^20^ and Personal Strength items of the PTGI^25^. We took care to avoid conceptually similar items to mitigate potential multicollinearity problems and ensure the discriminant validity of the COVID-19 CGA scale. We used Efklides’^28^ metacognitive theory, which includes affective metacognitive experiences, to modify and develop the items to capture self-awareness related to growth in character, resilience, and adaptability during the pandemic. For instance, Tedeschi and Calhoun’s^25^ original item capturing personal strength, “I discovered that I’m stronger that I thought I was”, was extended to two items capturing similar concepts but in relation to the pandemic and resilience: “Going through the lockdown made me realise that I’m stronger than I thought I was” and “Experiencing the pandemic has built my strength of character”. However, the item “I know I can handle difficulties” (from PTGI) was not included due to its conceptual similarity to CD-RISC items such as “I can deal with whatever comes my way.” Pivoting Efklides’^28^ theory on metacognitive experiences to posttraumatic growth and resilience, three additional items were added: “I have a better understanding of how resilient I am as a result of the pandemic experience”; “I have a better understanding of how to adapt to challenging situations as a result of the pandemic experience” and “The lockdown made me reflect on the type of person I am”. Participants were instructed to rate the items in relation to how they felt during the COVID-19 pandemic using a 7-point Likert scale from 'does not apply at all’ (0) to 'fully applies’ (6).

It should be noted that the COVID-19 CGA scale was not designed to replace the PTGI^25^. Instead, we pivoted posttraumatic growth theory to capture transient adaption to trauma induced by lockdown *amid ongoing pandemic*, not a long-lasting change that is captured by the PTGI *after* a traumatic event.

## Studies 1 and 2

**Fig. 1:**
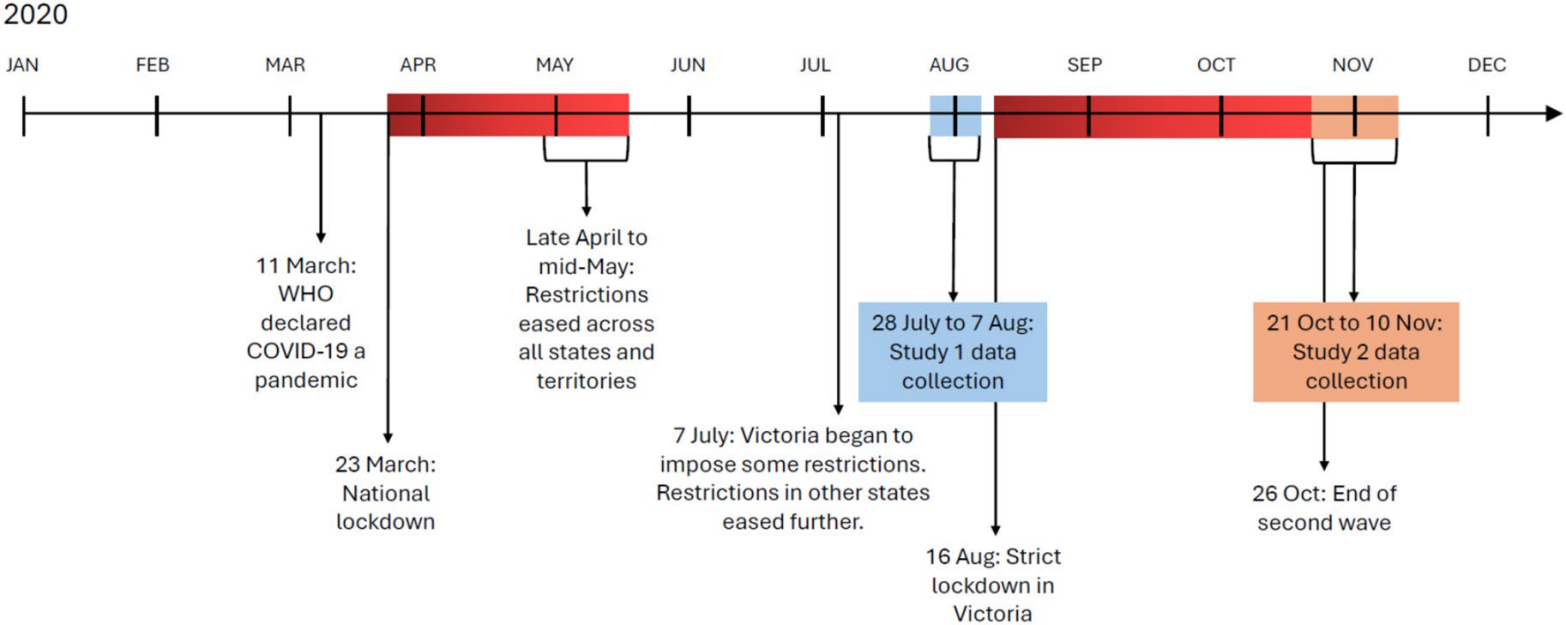
Rough timeline of key lockdowns and data collection periods in 2020.

We conducted two cross-sectional online surveys during two key emergency stages of the pandemic in Australia: after the first prolonged national lockdown (during recovery stage; Study 1), and towards the end of the second lockdown in one of Australia’s states (Victoria, where restrictions were easing and a return to normalcy was within sight; Study 2) to enable us to capture potential character growth at these two critical points. Relevant details are included in the Methods section at the end of this paper, and further details of Study 2 were published previously^2^. We specified outcome variables that captured different metrics of mental health, both positive functioning (mental well-being, Study 1) and negative functioning (psychological distress and anxiety, Study 2). The CF2 variables were key covariates of interest given the purpose of this research: to examine psychological constructs that predispose people to survive and thrive during the crisis (specific hypotheses are presented below). To aid the focus of this paper, we present both studies side by side. This comparative approach facilitates a better understanding of the similarities and differences between the studies. Figure 1 presents a rough timeline of key lockdowns and data collection periods. Details of lockdowns and restrictions for each state and territory are presented in Supplementary Material 3 (Tables S1, S2).

### Mental health outcome metrics

It has been increasingly recognised and empirically supported that mental health and mental illness are related but distinct dimensions^29,30^. That is, mental health is not merely the absence of psychopathology but exists on a separate continuum encompassing positive functioning. On the other hand, mental illness reflects symptoms of dysfunction. In this paper, we adopt this dual continuum framework to provide a comprehensive exploration of mental functioning and assess CF2 constructs and the newly developed COVID-19 CGA across diverse aspects of mental functioning.

In Study 1, which took place after the first-wave of national lockdown—a recovery stage (see Fig. 1)—we examined *mental well-being (MWB)*–defined as positive mental functioning, encompassing affective-emotional aspects, cognitive-evaluative dimensions, and psychological functioning^31^. We administered the Warwick-Edinburgh Mental Well-being Scale (WEMWBS) twice. The first time, it captured participants’ perceptions of their mental well-being post-lockdown, with a recall period of the last two weeks (July–August 2020), and the second time, the recall period was during the peak of the lockdown (mid-March to mid-May 2020). This yielded two outcome variables for modelling: (1) Mental Well-being (MWB, higher scores indicated better MWB) and (2) Mental Well-being Recovery (MWB recovery). The latter was calculated as the difference between participants’ post-lockdown MWB and their MWB during lockdown, and positive differences were interpreted as MWB recovery. To mitigate possible response distortion, the Social Desirability Scale was included in Study 1 and controlled for in all analyses.

In Study 2, we built on Study 1 by examining symptoms of mental dysfunction, namely, metrics of psychological distress and general anxiety collected during the final stages of the second-wave lockdown in some states (see Fig. 1). Lower scores on these measures reflect fewer symptoms of distress and anxiety, which may be indicative of either preservation of mental functioning despite the pandemic’s challenges or recovery from mental health impacts experienced earlier in lockdown.

### Covariates

To assess the associations of the psychological constructs of interest beyond other covariates of mental health, we controlled for demographic characteristics (age, biological sex, socioeconomic status, level of education, income, household number, financial comfort, social support, and physical health), individual differences (personality, cognitive abilities, attitudes, and political/cultural factors), and situational COVID-19 related factors (beliefs about COVID-19 protective measures and COVID-19 impact). We also controlled for social desirability in Study 1 to rule out the tendency to provide socially auspicious responses^32^. To preserve the focus of this paper, a brief literature review and hypotheses related to these covariates are presented in Supplementary Material 1.

### Aims and Hypotheses

Aims and Hypotheses in relation to the psychological constructs of interest are postulated in this section.

**Aim I**: To examine the factorial structure of the newly proposed COVID-19 CGA Scale.

- ***Study 1:*** We expected the items to converge in one factor using EFA (H1.1)
- ***Study 2:*** We expected CFA to support a one-factor model (H1.2).

**Aim II:** To examine the association between the target psychological constructs and mental health outcomes.

- ***Study 1:*** We expected impulsivity and lack of self-control to negatively predict variance in MWB (H2.1.1), while resilience and adaptability (H2.1.2) and CGA (H2.1.3) positively predict variance in MWB. We expected identical predictions for MWB recovery (H2.1.4, H2.1.5 and H2.1.6, respectively).
- ***Study 2:*** We expected Intolerance of Uncertainty to positively predict variance in psychological distress (H2.2.1) and anxiety (H2.2.3), and the CGA factor(s) to negatively predict variance in psychological distress (H2.2.2) and anxiety (H2.2.4).

## Method

### Participants and procedure

For multiple regression analyses, the suggested sample size is 15 to 20 observations per covariate^33^. Using this heuristic, a sample size of 345 to 460 was considered adequate for Study 1, as there were 23 covariates included in the models. In study 2, a sample size of 200 to 300 was considered adequate as there were 20 covariates included in the models. However, in Study 2, we recruited a much larger sample as this study was part of a larger project aiming to representatively capture different regions of Australia undergoing very different levels of restrictions during the second wave of the pandemic (explained further below, see also^2^ for details).

### Study 1

An Australian sample of 455 participants was recruited via Prolific (https://www.prolific.co/) to complete an online survey. Participants were compensated £5 (8.5 AUD) for their time. After data cleaning (see Supplementary Material 2), the final sample included 417 participants. The sample had a mean age of 34.38 (SD = 12.92, ranging between 18 and 73) and were 48.7% female. Reflecting good regional representation of the Australian population (see Australian Bureau of Statistics^34^), 31.4% were from New South Wales (NSW), 28.5% from Victoria—the two most densely populated states—and 40.1% from all other states collectively. The data cleaning protocol and detailed sample characteristics are outlined in Supplementary Material 2.

Data was collected from 28 July to 7 August 2020 when Australian national restrictions were eased everywhere except Victoria where gatherings were restricted, and the second-wave lockdown became imminent, first with specific postcode lockdowns and then with wider metropolitan lockdowns^35^. NSW, being another metropolitan centre of Australia, was faced with the possibility of another outbreak. Thus, the analyses contrasted Victoria and NSW with all other states. In study 1, all states and territories were similarly affected by the first wave of the pandemic as lockdown was national, thus sampling was representative of the population.

### Study 2

An Australian sample of 2007 participants were recruited via Toluna (https://au.toluna.com) to complete an online survey. Participants received Toluna ‘panel points’ to compensate for their time, equivalent to approximately 1AUD. Data was collected between 21 October and 10 November 2020. Participants were quota sampled by four regions based on the intensity of COVID restrictions and number of active cases^35^, see Supplementary Material 2 and^2^. Participants were then quota sampled by age and sex to be representative of the Australian general population. Each group comprised 25% of the sample. After data cleaning, the final sample of 1898 had a mean age of 47.43 (SD = 17.81, ranging between 18 and 86), and were 51.8% female. Full sample characteristics and data exclusions are presented in Supplementary Material 2.

### Measures

Measures employed are summarised in Tables 1 and 2, and full details of original COVID-19 measures are presented in Supplementary Material 4.

**Table 1:** Outcome and focal independent variables employed in Study 1 and Study 2.

Measure	Construct	Number of items and response scale	Dimensions and example items	Internal consistency (previous studies)
Outcome variables				
*Study 1*: Warwick-Edinburgh Mental Well-being Scale^31^	Mental well-being	14 items; (1) *none of the time* to (5) *all of the time*	“I was feeling relaxed” Participants rated each item twice, based on (1) how they felt in the past two weeks and (2) how they felt during the peak of the COVID-19 first-wave lockdown (mid-March to mid-May)	0.89^31^
*Study 2*: Kessler Psychological Distress Scale (Kessler-10;^46^)	Psychological distress	10 items; (1) *none of the time* to (5) *all of the time*	“During the past 30 days how often did you feel tired out for no good reason?”	0.93^46^
*Study 2*: Generalized Anxiety Disorder-7 (GAD-7;^47^)	Anxiety	7 items; (1) *not at all* to (4) *nearly every day*	“Over the last two weeks how often have you been bothered by the following problems?” E.g., “Feeling nervous, anxious or on edge”	0.92^47^
Independent variables				
CF2 (Study 1)				
Connor-Davidson Resilience Scale Short Version^20^	Resilience	10 items; (0) *not true at all* to (4) *nearly always true*	“I can deal with whatever comes.”	0.85^20^
Individual Adaptability Scale^18^	Adaptability	15 items; (1) *strongly disagree* to (5) *strongly agree*	Two subscales were included in this study: Handling Crises: “I am able to maintain focus during emergencies” Tolerance for Uncertainty: “I perform well in uncertain situations”	0.81 (Crises); 0.74 (Uncertainty)
Brief Self-Control Scale^11^	Self-control	13 items; (1) *not at all like me* to (5) *very much like me*	“I am good at resisting temptation”	0.83 and 0.85^11^
Short UPPS-P Impulsive Behavior Scale^10^	Impulsivity	20 items; (1) *disagree strongly* to (4) *agree strongly*	Emotion-Based Rash Action (positive and negative urgency): “I tend to lose control when I am in a great mood”; Sensation-Seeking: “I quite enjoy taking risks”; Deficits in Conscientiousness (Lack of Premeditation and Perseverance): “I finish what I start”	0.74 to 0.88^10^
COVID-19 Character Growth Awareness Scale	COVID-19 character growth awareness	5 items; *(0) does not apply* to (6) *fully applies*	“Going through the lockdown made me realise that I’m stronger than I thought I was.”	–
Intolerance of uncertainty (Study 2)				
Intolerance of Uncertainty Scale Short Form^48^	Intolerance of uncertainty	12 items; (1) *not at all characteristic of me* to (5) entirely characteristic of me	“Unforeseen events upset me greatly”	0.91^48^

**Table 2:** Covariates measures employed in Study 1 and Study 2.

Measure (authors)	Number of items and response scale	Dimensions and example items	Internal consistency (previous studies)
COVID-19 measures (Study 1 and 2)			
COVID-19 Beliefs (study-specific, see Supplementary Material 3)	7 items; (1) *strongly disagree* to (5) *strongly agree*	Response Efficacy Beliefs: “Social distancing is effective in slowing the spread of COVID-19” Perceived Barriers Beliefs: “Social distancing is destroying our economy”	–
COVID-19 Impact Index (study-specific, see Supplementary Material 3)	15 items; Sliding scale from − 10 (e.g., worse off) to + 10 (e.g., better off)	“What impact has COVID-19 had on your time availability?”	–
Multidimensional COVID-19 Worry Scale (study-specific, see Supplementary Material 3)	21 items (1) *never* to (4) *always*	Personal and Family Concerns: “I am worried about my health due to COVID-19”; Infrastructure/Supplies Concerns: “I am worried about grocery stores running out of food and/or other supplies”; Economy/Liberties Concerns: “I am worried about political systems failing”; Personal Financial Concerns: “I am worried about my financial situation”	
Other (Study 1)			
Mini International Personality Item Pool^37^	20 items; (1) *very inaccurate* to (5) *very accurate*	Extraversion: “I am the life of the party”; Agreeableness: “I sympathize with others’ feelings”; Conscientiousness: “I get chores done right away” Neuroticism: “I have frequent mood swings”; Intellect/Openness: “I have a vivid imagination”	0.65 to 0.82^37^
Marlowe-Crowne Social Desirability Scale Short Form^49^	13 items True/False response	“I’m always willing to admit it when I make a mistake”	0.76^49^
Demographics			
Study 1: Social Support Scale (adapted from^50^,Supplementary Material 3)	6 items	“How many people were close enough to you that you could count on them if you had COVID-related mental health issues [e.g., isolation, worries/anxiety]?”	0.64 for the original scale^51^
Physical Health Questionnaire^52^	14 items; (1) *not at all* to (7) *all of the time*	“How often have you woken up during the night”	0.61 to 0.88^52^

Study 1: age (in years), biological sex (0 = male, 1 = female), level of education (1 = none; 2 = primary school; 3 = high school diploma/certificate; 4 = vocational/trade certificate; 5 = associate degree; 6 = bachelor’s degree; 7 = master’s degree; 8 = doctorate degree), income (1 = less than $20,000; 2 = $20,000-$34,999; 3 = $35,000-$49,999; 4 = $50,000-$74,999; 5 = $75,000-$99,999; 6 = $100,000-$149,999; 7 = $150,000-$199,999; 8 = $200,000 or more), number of people in the household (as stated), financial comfort (rated on a sliding scale from 0 to 100), state and postcode. The coding system for level of education and income, and occupational status was consistent with those in the Australian census.

Study 2: age (in years), biological sex (0 = male, 1 = female), level of education (1 = year 11 and below; 2 = year 12; 3 = trade certificate/apprenticeship; 4 = diploma; 5 = bachelor’s degree; 6 = higher degree), chronic comorbidities, health risk factors, and financial comfort (0 = not at all comfortable; 10 = completely comfortable), state of residence, Australian citizenship/permanent residency status (0 = no; 1 = yes), Aboriginal or Torres Strait Islander (0 = no; 1 = yes).

### Statistical analyses

**Fig. 2:**
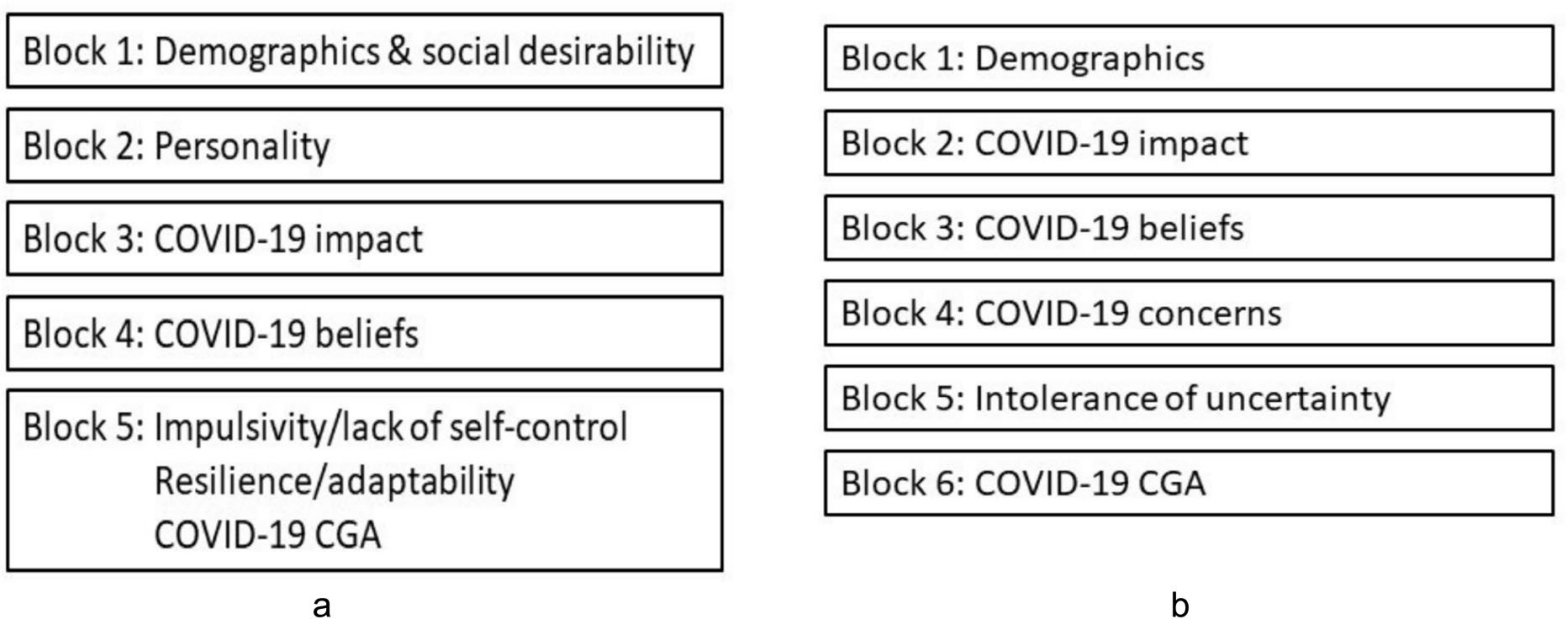
(**a**) Hierarchical regression model for Study 1. (**b**) Hierarchical regression model for Study 2.

We first examined the psychometric properties of the COVID-19 CGA scale, using Exploratory and Confirmatory Factor Analyses (EFA and CFA) in Studies 1 and 2, respectively. Then, we performed a series of hierarchical multiple-regression analyses. Figure 2a presents the models predicting mental well-being and recovery in Study 1. Figure 2b presents the models predicting psychological distress and anxiety in Study 2. The constructs of interest were included in the last block to determine their predictive validity above and beyond other known covariates. Their stability and time precedence determined the order of auxiliary covariates. All analyses were conducted in SPSS and AMOS (*v*26; IBM Corp.^36^).

## Results

### Factor analyses

#### COVID-19 character growth awareness (COVID-19 CGA) scale

Correlations between the items ranged between 0.50–0.82 in Study 1 and between 0.54–0.80 in Study 2 (Table S3 in Supplementary Material). In Study 1, the scree plot suggested one factor with an eigenvalue greater than one, explaining 73.05% of the variance. In Study 2, a one-factor model was fitted (*χ*^2^_5_ = 123.57). Its fit indices were: CFI and GFI = 0.98, TLI = 0.96, RMSEA = 0.11 with 90% CI (0.10; 0.13), RMR = 0.08. The model was re-run allowing the error terms for items 1 and 2 to correlate given that they both refer to self-reflection after lockdown. The correlation between the error terms was 0.22 (*p* < 0.001). The model returned excellent fit: *χ*^2^_4_ = 45.48; CFI and GFI = 0.99, TLI = 0.98, RMSEA = 0.07 with 90% CI (0.06; 0.09), RMR = 0.04.

Factor analysis results are presented in Table 3. Supporting H1.1 and H1.2, all items loaded strongly onto one factor with factor loadings ranging from 0.79 to 0.91 in Study 1 and 0.63 to 0.90 in Study 2, and communalities ranging from 0.62 to 0.83 in Study 1 and 0.40 to 0.81 in Study 2. The COVID-19 CGA scale returned excellent reliability estimates of 0.90 in both studies. The factor score (Bartlett method) was saved and used as an independent variable in further analyses in both studies.

**Table 3:** Factor loadings and communalities for COVID-19 Character Growth Awareness Scale.

	Study 1 (N = 417)					Study 2 (N = 1898)			
	M	SD	FL	*h*^2^		M	SD	FL	*h*^2^
1. Going through the lockdown made me realise that I’m stronger than I thought I was	3.60	1.67	0.79	0.62		3.73	1.65	0.73	0.53
2. The lockdown made me reflect on the type of person I am	3.64	1.77	0.80	0.65		3.58	1.71	0.63	0.40
3. I have a better understanding of how resilient I am as a result of the pandemic experience	3.97	1.52	0.88	0.77		4.03	1.54	0.81	0.66
4. Experiencing the pandemic has built my strength of character	3.72	1.62	0.91	0.83		3.76	1.66	0.90	0.81
5. I have a better understanding of how to adapt to challenging situations as a result of the pandemic experience	3.94	1.56	0.89	0.79		3.96	1.58	0.89	0.79

M = mean; SD = standard deviation; FL = factor loading.

### Descriptive statistics, reliabilities, and correlations

Descriptive statistics and Cronbach’s alpha for all measures are presented in the Supplementary Material (Tables S4 and S5) and were comparable to previous research (e.g.,^4,37^). Correlations between continuous variables in each study are also presented in the Supplementary Material (Tables S6 and S7). Resilience and adaptability metrics correlated strongly and positively in Study 1 (between 0.65–0.69¸ p < 0.001) and were combined into a composite (Bartlett method), labelled Resilience/Adaptability. It captured 78.3% of the variance, with factor loadings between 0.88 and.89, and communalities between 0.77 and 0.80. Similarly, measures of impulsivity and lack of self-control shared albeit weaker a positive manifold (r’s range between 0.20–0.59, p’s < 0.01). The relevant scores were also combined into one composite (Bartlett method), labelled Impulsivity/Lack of Self-control. The composite captured 53.05% of the variance, with factor loadings ranging between 0.56 and 0.83 and communalities between 0.31 and 0.69.

Independent samples t-tests examining the relationships between demographics and the dependent variables are reported in Supplementary Material 5.

### Regression Analyses

Each model’s assumptions were checked and, with minor caveats, satisfied. In Study 2, the linearity assumption for intolerance of uncertainty was violated. A quadratic term was also included in the model to remedy this issue. Variance inflation factor and tolerance estimates indicated no issues with multicollinearity. To maintain the paper’s focus, we will address results relevant to the research’s focus while briefly reporting on the other results. Full regression results, including multicollinearity metrics, are presented in Supplementary Material Tables S8–S13.

#### Study 1: Mental well-being (MWB)

Table 4 presents the results of the regression analysis predicting variance in MWB. The regression model predicted 56.9% of the variance in MWB, with block 5 adding 6% (*p* < 0.001). Supporting H2.1.1, H2.1.2 and H2.1.3, Impulsivity/Lack of Self-Control, Resilience/Adaptability and COVID-19 CGA were significant covariates (negative, positive and positive) of MWB in the multivariate model beyond the previous blocks and incrementally to each other in the final model.

**Table 4:** Regression models predicting mental well-being post-lockdown and recovery in mental well-being (from lockdown to post-lockdown), Study 1 (N = 417).

Predictor	DV: Mental Well-being			DV: Mental Well-being Recovery		
	*β*	R^2^	ΔR^2^	*β*	R^2^	ΔR^2^
Block 1		0.34	0.34***		0.05	0.05*
Age	0.04			− 0.10		
Sex	− 0.02			0.06		
Victoria	− 0.03			− 0.12*		
NSW	− 0.01			− 0.04		
Educational attainment	0.01			− 0.04		
Annual income	0.05			− 0.02		
Household number	0.04			0.10		
Physical symptoms	− 0.22***			0.06		
Financial comfort	0.18***			0.08		
Social support	0.33***			− 0.02		
Social desirability	0.20***			0.03		
Block 2		0.48	0.14***		0.10	0.05**
Extraversion	0.16***			0.21***		
Agreeableness	0.11*			− 0.08		
Conscientiousness	0.07			0.17**		
Neuroticism	− 0.33***			0.07		
Intellect	0.10*			0.00		
Block 3		0.51	0.03***		0.12	0.02*
Positive COVID-19 impact	0.16***			0.13*		
Negative COVID-19 impact	− 0.09*			0.09		
Block 4		0.51	0.01		0.12	0.00
Response efficacy beliefs	− 0.07			− 0.02		
Perceived barriers beliefs	0.00			0.00		
Block 5		0.57	0.06***		0.15	0.03**
Impulsivity/lack of self control	− 0.17**			0.03		
Resilience/adaptability	0.20***			− 0.03		
COVID-19 CGA	0.16***			0.20***		

*β* = standardised regression coefficient from each hierarchical block of variables. *p < 0.05; **p < 0.01; ***p < 0.001.

*Control variables* The variables in block 1 predicted the highest amount of variance in MWB (34%). Available social support was the strongest covariate, followed by physical symptoms as a strong negative covariate, and social desirability and financial comfort as strong positive covariates (*p-*values < 0.001). No other demographics were significant. In block 2, Neuroticism was the strongest negative covariate (*p* < 0.001), while Extraversion, Agreeableness (*p-*values < 0.01) and Intellect (*p* < 0.05) were positive covariates. In block 3, positive COVID-19 impact was a positive covariate (*p* < 0.01), whereas negative COVID-19 impact was a weaker negative covariate (*p* < 0.05). In block 4, neither of the belief variables significantly associated with MWB.

#### Study 1: MWB recovery

Table 4 also presents the hierarchical regression analysis results of recovery in MWB (higher positive scores indicate better recovery). The regression model predicted 15.1% of variance. This estimate is notably lower than in the other models, albeit still significant and meaningful. In block 5, COVID-19 CGA was the only significant covariate of MWB recovery (*p* < 0.001), with Impulsivity/Lack of Self-Control and Resilience/Adaptability being non-significant. Thus, only H2.1.6 was supported.

*Control variables* In block 1, living in Victoria or NSW compared to other states was a significant, albeit weak, negative covariate (*p* < 0.05). In block 2, Extraversion and Conscientiousness were positive covariates (*p* < 0.001 and *p* < 0.01). In block 3, positive COVID-19 impact was a weak positive covariate (*p* < 0.05). Neither of the belief variables were significant in block 4.

#### Study 2: Psychological distress

Table 5 presents the results of the hierarchical regression analysis of Kessler-10. The model predicted 54.9% of the variance (*p* < 0.001). Both the linear and square intolerance of uncertainty variables were positive covariates supporting H2.2.1 (*p* < 0.001). Replicating the results of Study 1 and supporting H2.2.2, COVID-19 CGA was a negative covariate over and above all other variables (*p* < 0.001).

**Table 5:** Regression models predicting psychological distress and generalized anxiety, Study 2 (N = 1898).

Predictor	DV: Distress			DV: Anxiety		
	*β*	R^2^	ΔR^2^	*β*	R^2^	ΔR^2^
Block 1		0.25	0.25***		0.22	0.22***
Age	− 0.38***			− 0.35***		
Sex	0.06**			0.06**		
Victoria	− 0.01			0.01		
NSW	− 0.05*			− 0.04		
Australian citizenship/residency	0.02			0.01		
Aboriginal/Torres Strait Islander	0.01			0.01		
Educational attainment	0.02			0.01		
Chronic comorbidities	0.18***			0.15***		
Health risk factors	0.11***			0.12***		
Financial comfort	− 0.23***			− 0.22***		
Block 2		0.36	0.10***		0.33	0.11***
Positive COVID-19 impact	0.08***			0.08***		
Negative COVID-19 impact	0.36***			0.37***		
Block 3		0.37	0.02***		0.34	0.01***
Response efficacy beliefs	− 0.00			0.00		
Perceived barriers beliefs	0.13***			0.10***		
Block 4		0.43	0.06***		0.40	0.06***
Personal/family concerns	0.23***			0.23***		
Infrastructure/supplies concerns	0.05			0.04		
Economy/liberties concerns	− 0.06*			− 0.03		
Personal financial concerns	0.11***			0.11***		
Block 5		0.54	0.11***		0.50	0.10***
Intolerance of uncertainty (IU)	0.35***			0.33***		
IU square term	0.09***			0.09***		
Block 6		0.55	0.01***		0.51	0.01***
COVID-19 CGA	− 0.11***			− 0.09***		

*β* = standardised regression coefficient from each hierarchical block of variables. *p < 0.05; **p < 0.01; ***p < 0.001.

*Control variables* In block 1, age and financial comfort were negative covariates (*p* < 0.001). Greater comorbidities and health risk factors were positive covariates (*p* < 0.001). Not residing in NSW was also a weak covariate (*p* < 0.05). In block 2, both impact variables were significant positive covariates of distress (*p* < 0.001), with the negative impact being considerably stronger. In block 3, only perceived barriers beliefs were a significant positive covariate (*p* < 0.001). In block 4, concerns about self/family and finances were positive covariates (*p* < 0.001), and economy/liberties concerns was a weak negative covariate (*p* < 0.05).

#### Study 2: Anxiety

Table 5 also presents the results of the regression analysis of GAD-7 scores. The final model was significant and predicted 50.6% of the variance (*p* < 0.001). Both intolerance of uncertainty linear and square terms were significant in block 5, supporting H2.2.3 (*p* < 0.001). Replicating results of Study 1, and supporting H2.2.4, COVID-19 CGA was a negative covariate above and beyond all other variables in the model (*p* < 0.001).

*Control variables* In block 1, age was a strong negative covariate (*p* < 0.001). Lower ratings of financial comfort, having greater comorbidities and health risk factors were significant covariates (*p* < 0.001), and being female was a weak covariate (*p* < 0.01). In block 2, negative COVID-19 impact was a strong positive covariate, and positive impact was a weaker covariate, also positively (*p* < 0.001). Perceived barriers, personal/family and financial concerns were positive covariates in blocks 3 and 4, respectively (*p* < 0.001).

## Discussion

There is limited evidence of the validity of the constructs proposed by the Cognitive Fitness Framework (CF2^8^) in the general population. The two studies presented in this paper aim to address this gap by examining the applicability and generalizability of findings across diverse populations in Australia during the emergency stages of the COVID-19 pandemic. The emergency stages of the pandemic provided a unique, ecologically valid opportunity to assess relationships between psychological constructs and individuals’ capacity to handle changes, prolonged uncertainty, and challenging conditions. Thus, we examined key psychological constructs outlined in the CF2, including impulsivity/lack of self-control and resilience/adaptability^8^ and their associations with individuals’ ability to thrive in high-risk settings. We extended the investigation to intolerance of uncertainty and self-reflections of personal growth to deepen our understanding of the psychological constructs that might contribute to positive adaptation under challenging and uncertain circumstances.

In two studies, we examined different aspects of mental functioning during the pandemic’s emergency stages, incorporating a novel tool capturing awareness of character growth arising from the pandemic. The COVID-19 CGA scale was developed based on integrating three theories—posttraumatic growth, resilience, and metacognition—as a proof of concept, with an initial examination of its factorial structure, reliability, and predictive validity. Together, these findings inform and extend cognitive fitness and resilience theories, offering insights into the psychological factors that contribute to adaptation in the face of crisis. Below, we discuss results addressing the CF2 constructs and the newly proposed COVID-19 CGA construct. To preserve the focus of this paper, a discussion of results related to control variables can be found in Supplementary Material 7.

### CF2 and other psychological constructs

In Study 1, Impulsivity/Lack of Self-Control and Resilience/Adaptability emerged as important covariates of mental well-being (MWB). Supporting the CF2 model^8^, lower impulsivity and greater resilience/adaptability were associated with maintaining better MWB following the first wave of the pandemic. In Study 2, replicating and extending results of Andrews and colleagues^38^, the linear term of intolerance of uncertainty accounted for a significant percentage of variance in psychological distress and anxiety. Additionally, the quadratic term indicated an exponential relationship between intolerance of uncertainty and both distress and anxiety, which was not hypothesized. However, it clarifies the nature of the relationship, such that as intolerance of uncertainty increases, so does distress and anxiety, but the rate of increase diminishes at a certain level. This result is novel, and given a somewhat weak quadratic term, needs to be replicated before making substantial interpretations.

The new COVID-19 CGA scale was developed to capture perceptions and awareness of how one has grown to become more resilient through challenges, specifically experiencing the pandemic. The items converged into one robust factor using both Exploratory and Confirmatory Factor Analyses, had high internal consistency, and demonstrated excellent construct and predictive validity indices. The construct validity of this scale was evaluated through correlations with other measures. It shared no or weak relationships with personality dimensions and, importantly, social desirability, attesting to the authenticity of responses. It shared moderate positive correlations with positive COVID-19 impact and Resilience/Adaptability. All relationships were in the expected directions and of no more than moderate strength, supporting the scale’s discriminant and convergent validity.

COVID-19 CGA also demonstrated utility in predicting variance in positive adaptation during COVID-19. It was a potent covariate, predicting a substantial amount of variance in post-lockdown MWB levels. Notably, although it is not currently proposed as part of the CF2 model, it was the only significant covariate of positive changes in MWB levels during the post-lockdown recovery period in the final block containing two proposed CF2 constructs, Impulsivity/Lack of Self-Control and Resilience/Adaptability. Study 2 clarified this prediction of variance in psychological distress and generalized anxiety, utilising some of the same and some additional covariates. Replicating and extending Study 1, COVID-19 CGA was associated with lower psychological distress and anxiety levels above and beyond all other variables. These findings attest to the robustness and generalizability of the Study 1 findings to different mental health metrics, and a new sample tested during a different period of the pandemic. The results also inform and extend resilience theory, suggesting that recognizing that experiencing challenges makes one stronger contributes to recovery or bouncing back in the face of adversity.

In Study 1, COVID-19 CGA predicted variance in both outcome metrics above and beyond the Resilience/Adaptability composite, which was captured through traditional measures of resilience and adaptability as robust capacities. These findings demonstrate that awareness of one’s growth shares important relationships with positive outcomes and recovery beyond stable individual differences in resilience and adaptability in the context of the pandemic. The finding that resilience predicted variance in MWB but not its recovery supports the view that trait resilience is associated with maintenance of functioning levels rather than bouncing back.

The inclusion of the COVID-19 CGA scale captures resilience beyond the trait approach, acknowledging the context-specific nature of the resilience process (inherently involving traits and outcomes), which aligns with existing theories of resilience (e.g.,^39,40^). Extending these models, this research highlights a previously overlooked aspect of the process—self-reflection and appraisal of how going through challenges contributes to the growth of resilience. However, these results also suggest that there are robust differences in perceptions of growth, suggesting that there is value in further study of them to understand their antecedents (e.g., previous experiences, early childhood experiences and general trauma).

Overall, these findings inform theories of resilience by demonstrating the potential important role of self-reflections in maintaining mental health during prolonged challenges, bouncing back, and possibly, building resilience. Future studies are needed to provide further validation of this construct and to develop a generic measure that can be adapted to different contexts to examine its role in positive adaptive outcomes during crises in other domains, including the military. The results may also have implications for measuring resilience (see^41^ for a review).

This finding is also important in applied settings as unlike impulsivity which is understood to be a stable construct^42^, resilience has been argued to be malleable, changing in the face of challenges, in this case, the pandemic. Thus, in applied settings, targeting these beliefs of character growth through facing challenges may provide a pathway to foster recovery via building resilience. Both of our studies were cross-sectional, so our inferences are correlational rather than causal. Future research could investigate this point using experimental and longitudinal designs.

### Strengths and limitations

We captured a comprehensive range of constructs demonstrated to be associated with mental health during COVID-19 in the emerging literature. They included demographic and situational factors (e.g., sex, age, occupation/employment status, social support, see^43^ for a review), fears/concerns associated with COVID^4^ and perceptions about regulations (e.g.,^4,44,45^). In all analyses, we controlled for these key constructs and social desirability before examining the constructs of interest. Thus, these results support the importance of the newly developed construct of awareness of character growth through the pandemic, the only variable that emerged as a covariate of all four dependent variables across both studies. However, given the cross-sectional nature of this research, no causality is implied, and further longitudinal investigation is needed to examine the directionality of the relationships. Importantly, this scale shared a minimal relationship with the social desirability scale, supporting the authenticity of responses.

Due to logistical constraints on survey duration, we did not examine relationships between the new COVID-19 CGA Scale and other related measures, such as the Post-Traumatic Growth Inventory (PTGI). It should be noted, however, that the COVID-19 CGA scale, developed within the Cognitive Fitness Framework, complements rather than replaces the PTGI, focusing on character growth, resilience and adaptability in the unique context of the pandemic, with future studies needed for broader validity testing, including the CGA Scale’s relationship to the PTGI in diverse settings.

With some caveats (over-representation of young and educated people), we collected data from a reasonably representative sample of people in Australia, achieving good power and generalizability within that sample. However, the sampling method may still produce potential selection bias, as do any sampling strategies of general populations, although these biases may differ by method. Nevertheless, we captured a heterogeneous sample, varying in age, sex, education, pre-existing health conditions, states of residency and economic situation, and this variability was a key requirement for our purposes of describing associations between constructs rather than estimating the prevalence of any particular construct.

To control the survey’s length, we used brief versions of scales, and their psychometric properties may have been impacted. However, most measures had reasonable to excellent reliability estimates. Perceived Barrier Beliefs had a low reliability estimate of 0.54, likely due to the low number of items. Further development and refinement are required for future use. The strength of this research lies in capturing positive and negative aspects of mental health outcomes at different stages of the pandemic, as it allowed us to assess the replicability and generalizability of our findings.

Future research could examine alternative explanations, e.g. that the associations between CF2 constructs and adaptation might be partly due to shared variance influenced by common external stressors. In other words, these constructs may not directly contribute to adaptation, but rather they all fluctuate together in response to acute stress, such as the uncertainty and challenges presented by the pandemic. Moreover, character growth and awareness may just be temporary shifts that might not necessarily represent individuals’ long-term capacities for adaptation or resilience but rather short-term adjustments to specific, unprecedented demands of the pandemic. This, however, is unlikely due to the robustness of our findings in the two current studies based on different populations and different stages of pandemic. Still, longitudinal research would be valuable to clarify this point.

Future pandemics most likely present similar challenges as the COVID-19 pandemic, and if necessary, this instrument can be modified moving forward. While tailored to the COVID-19 pandemic, the CGA scale can be modified for use in other transient crises characterised by significant social, economic, or environmental challenges, such as large-scale technological disruptions or geopolitical instability.

## Conclusion

Supporting the CF2 model^8^, these results highlight the importance of cognitive fitness constructs, including impulse control, resilience, adaptability, and the newly proposed awareness of character growth to adapt and recover quickly under demanding, uncertain conditions, signalling preparedness to face further challenges. The results also support the importance of intolerance of uncertainty as a key covariate of psychological health. The COVID-19 CGA scale was a potent positive covariate predicting significant percentage of variance in mental well-being recovery, demonstrating its utility beyond the already established CF2 constructs of impulsivity, self-control, resilience, and adaptability. The emerging importance of this construct helps to unify the theory of resilience as a trait, a process, and an outcome. The construct is also a promising candidate for future validation studies and potential inclusion in the CF2 and other models focusing on performance and sustained thriving in high-risk settings. The measure, however, would have to be extended and adjusted to reflect the relevant context (crises, natural disasters, battlefield). Studies of mental health and quality of life in the general population can also be informed by the CF2 model.

## Supplementary Information

Supplementary Information.

## Data Availability

Data for both studies is deposited in the Open Science Framework: https://osf.io/wdqfp/.
