# Supplementary material for: Cognitive fitness and mental health outcomes following COVID-19 lockdowns in Australia
Source: Sci Rep. 2025 Dec 25;16:89. doi: 10.1038/s41598-025-29205-w (PMC12764950; doi:10.1038/s41598-025-29205-w)
Supplement: Supplementary file 1 — Supplementary Information. [file 41598_2025_29205_MOESM1_ESM.docx]

Supplementary Material

“Cognitive Fitness and Mental Health Amidst Lockdown Dynamics in Australia”

*Scientific Reports*

Sabina Kleitman^1*^, Dayna J. Fullerton^1^, Lisa M. Zhang^1^, Madeleine T. King^1^, Eugene Aidman^1,2,3^

^1^School of Psychology, University of Sydney, NSW, Australia

^2^Divison of Human and Decision Sciences, Defence Science and Technology Group, Edinburgh, SA, Australia

^3^School of Biomedical Sciences and Pharmacy, University of Newcastle, NSW, Australia

*Corresponding author

E-mail: [sabina.kleitman@sydney.edu.au](mailto:sabina.kleitman@sydney.edu.au)

**1. Covariates**

**Personality**

Personality traits have a long-standing role in the research of subjective and mental well-being (see Steel et al., 2008 for a review). Regarding mental well-being, high extraversion and low neuroticism have been shown to be robust personality predictors of aspects of mental well-being (e.g., Anglim & Grant, 2016; Grant et al., 2009; Keyes et al., 2002; Steel et al., 2008). Conscientiousness, intellect/openness, and agreeableness have shown less consistent, albeit still meaningful, relationships with different aspects of psychological well-being (e.g., Anglim & Grant, 2016; Steel et al., 2008). In their meta-analysis, Steel and colleagues (2008) demonstrated that openness to experience was related to positive affect and happiness, and conscientiousness was a strong positive predictor of quality of life, but not other aspects of subjective well-being.

The emerging COVID-19-related literature largely replicates these results, with high neuroticism relating to lower well-being during lockdown (Gubler et al., 2021). However, the relationship between extraversion and mental well-being during a pandemic might be more complex due to the socially isolating aspect of lockdowns. While extraverts have tendencies to exhibit and steadily maintain their naturally high positive affect, these tendencies might be affected by prolonged periods of isolation, thus changing the nature of the relationship between mental well-being and this personality dimension. It is currently unclear how extraversion is related to recovery in mental well-being following lockdown. Hampshire et al. (2021) demonstrated that ‘self-security’ and ‘conscientiousness’ were associated with reduced scores on self-perceived pandemic impact. Similarly, it is unclear how conscientiousness and neuroticism are related to the recovery of mental well-being after lockdown.

Thus, to clarify the emerging results we included a measure of the Big Five traits to account for the role of personality in mental well-being and its recovery during different stages of pandemic. Our expectations are consistent with the emerging findings outlined above. Including personality variables also allowed us to examine the discriminant validity of the CF2 constructs, including the newly proposed COVID-19 Character Growth Awareness variable.

**COVID-19 Related Factors**

The impact of the pandemic on individual’s daily lives has been widely varied. Whilst some lost their jobs, others undertook increased workload, and some experienced little change (e.g., see Coulombe et al., 2020 for a review). The same pattern can be expected for various aspects of life such as home life and family responsibilities, social life, mental health, physical health, and daily behaviours such as sleep, nutrition, physical activity, and time available for rest and hobbies. However, research examining the relationship between the impact of COVID-19 and mental wellbeing is sparse. Emerging research has reported significant negative effects of COVID-19 on mental health and is beginning to investigate the main causes of these effects and how they might differ between subpopulations (see Abbott, 2021).

As no suitable, validated measure of the impact of COVID-19 on daily life was available at the time of this research, we included a newly developed measure capturing perceptions of positive and negative impacts. The scale spans the key areas of life affected by the pandemic, including job security, financial comfort, changes to nutrition, exercise, sleep patterns, family dynamics/relationships, physical and mental health, social environment/connections, living space, time availability, routine, productivity, substance use, and loneliness (e.g., see Coulombe et al., 2020; Gangopadhyaya & Garrett, 2020 for reviews). We expect that the degree of impact COVID-19 has had on one’s life will play a critical role in mental well-being, with more negative impacts harming mental well-being, and vice versa for positive impacts.

**Demographics and Social Desirability**

Socio-demographic factors are well-documented predictors of mental health (e.g., Isaacs et al., 2018, World Health Organization & Calouste Gulbenkian Foundation, 2014). Thus, in addition to trait factors, we considered a range of demographic and situational factors which might influence how one responds emotionally to the COVID-19 pandemic. These included gender, age, level of education, income, number of people in the household, financial comfort, social support, and physical health. The emerging results based on about 380K participants from the UK during 2020 revealed larger differences in pre- to peak-UK lockdown mood assessment for specific groups, including older adults and participants with lower incomes (Hampshire et al., 2021). The authors suggest that relevant population factors “explain variance in mental health and can be statistically predicted from age, demographics, home and work circumstances, pre-existing conditions, maladaptive technology use and personality traits (e.g., compulsivity)” (Hampshire et al., 2021, p. 1). A recent systematic review also found that being female and having poorer physical health were associated with greater impact of the pandemic on psychological well-being (Vindegaard & Benros, 2020). Available support is also an important resource for mental well-being (see American Psychological Association, 2019).

We expect to replicate these findings in the present research whilst using these key demographics as control variables when considering the predictive role of the CF2 constructs on mental well-being and post-lockdown recovery. We also controlled for social desirability—the tendency of participants to give socially desirable rather than honest responses. This control is necessary to statistically rule out the possibility that responses were influenced by this pervasive response distortion tendency.

**2. Data Cleaning, Sample Characteristics, and Additional Method details**

**Study 1**

***Data Cleaning***

An Australian sample of 455 participants was recruited via Prolific online research engine. Fourteen people consented but failed to complete the survey. Twenty-four additional participants (5.4%) were excluded based on failing three or more out of five attention checks (2 people), providing a non-serious attempt (1 person), or completing the survey in an unreasonably short time (less than 10 minutes, 21 people). The demographic characteristics of participants removed from the final sample were similar to those retained, with one notable exception—more males (16) than females (8) were among the 25 removed respondents. This, however, did not change the overall gender ratio in the sample.

***Final Sample Characteristics***

The final sample of 417 had a mean age of 34.38 (SD = 12.92, ranging between 18 and 73) and were 48.7% female. There were no missing values, except for the sex of 3 participants (<1%) who responded ‘other’ and 4.3% for annual income, where participants indicated they preferred not to list their income. Given these small percentages, these responses were coded as missing values and treated with pairwise deletion in all analyses.

Reflecting good regional representation of the Australian population, 31.4% were from NSW, 28.5% from Victoria—the two most densely populated states—and 40.1% from all other states collectively. These proportions are comparable to Australian population statistics (see Australian Bureau of Statistics, 2022b). Similarly, reflecting the heterogeneous multicultural nature of the Australian society, participants came from a total of 46 different birth countries with the majority, 57.8%, born in Australia. The next most common birthplaces were the United Kingdom (5.3%), United States (2.9%), India (2.6%), and the Philippines (2.6%). The birth countries of the overseas-born participants in our sample are largely consistent with the most common birth countries of Australia’s overseas-born population reported by the Australian Bureau of Statistics (2022a). For nationality, 73.1% reported Australia as their nationality. The next most frequent nationalities were the United Kingdom (3.6%), India (2.6%), the United States (1.9%), and New Zealand (1.7%). About 33% of the sample had a High School Diploma or Vocational/Trade Certificate. An additional 47.5% had an Associate/Bachelor degree, and 19.2% reported having Master/Doctorate degrees. The reported levels of annual income varied between less than 20k (24.7%), 20-35k (13.2%), 25-50k (11.8%), 50-74k (20.4%), 75-99k (13.7%), 100-149k (6.7%), 150-199k (3.4%) and 200k or more (1.9%). The listed occupations included a broad range: student (21.6%), professional (e.g., scientist, architect/engineer, accountant, medical/health professional, teacher, musician; 26.9%), associate professional/transport (e.g. technician, hospitality/shop manager, sports coach, transport; 3.5%), manager/administrator (6.5%), tradespersons and related (1.7%), advanced clerical and service worker (e.g. secretary/personal assistant, flight attendant; 3.6%), intermediate clerical (e.g., receptionist, bank worker, carer, hospitality worker, dental assistant, beauty therapist; 9.6%), elementary clerical, sales and services (2.6%), labourers and related workers (2.2%), self-employed (5.3%), retired (4.1%), and unemployed (12.5%). This demonstrates that this sample of the Australian population has a good representation of ages, biological sex, socioeconomic status (income, education, occupations), countries of origin and ethnicity.

***Additional Method Details***

Given the nature of the survey, it was important that the order of the questionnaires did not affect responses. Thus, variables most likely to be affected by COVID-19-related responses were given first. They include resilience, adaptability, impulsivity/self-control, attitudes, and COVID-19 character growth awareness. These were followed by COVID-19 related questions about impacts and beliefs, followed by reports of demographic characteristics and well-being measures (physical and mental). Personality measures were given last as they captured stable traits. Moreover, to further mitigate priming effects, the dependent variable measures were separated from the focal independent variables by control measures e.g., demographic questions. This was done to reduce the tendency to use previous answers to inform subsequent answers where items are measuring related constructs, as the separation gives a chance for this information to leave the participant’s short-term memory. Several other measures were given to participants as part of a larger study. They are outside of the scope of this research and are described in other forthcoming paper(s) devoted to this research.

**Study 2**

***Sampling***

An Australian sample of 2007 participants were recruited via Toluna. Participants were quota sampled by four regions based on the intensity of COVID restrictions and number of active cases (Evershed et al., 2020). That is Metropolitan Melbourne (group 1) had the highest number of cases and had been under Stage 4 restrictions in August and September which continued to ease through the data collection period. Regional Victoria (group 2) had the next highest number of cases and had been under Stage 3 restrictions since August, easing just before data collection. NSW and Queensland (group 3) experienced similar numbers of cases and levels of restrictions in 2020, so formed the third group. All other remaining states and territories (group 4) had the lowest case numbers and the least restrictions.

***Data Cleaning***

Seven data quality checks were used to identify poor quality responses: six logic checks based on inconsistent responses and survey completion time less than 7.79 minutes which is equivalent to 30% of the median completion time. A total of 99 participants who failed two or more data quality checks were excluded. An additional 10 participants who were identified as speedy completers were excluded, resulting in a final sample of 1898 participants.

***Final Sample Characteristics***

The final sample had a mean age of 47.43 (SD = 17.81, ranging between 18 and 86), and were 51.8% female. Majority of the sample (77%) were born in Australia, 4.8% England, 1.9% New Zealand, 2.5% India, 0.6% Italy, 0.8% Vietnam, 1.7% Philippines, and 10.6% other. A large majority were also Australian citizens (86.2%) or permanent residents (9%). About 85% of the sample had completed Year 12 or higher, with 16.6% reporting Year 12 as their highest level of education completed, 17.3% Trade Certificate, 14% diploma, 25.8% Bachelor degree, and 11.4% reported having a Higher degree. Over half (57.5%) reported being employed, 12% unemployed and 30.5% not in the labour force. Employed participants worked in a range of industries: education and training (12.3%), retail (9.0%), health care and social assistance (8.8%), administrative and support services (8.5%), professional, scientific and technical services (8.3%), agriculture, forestry, fishing (7.1%), construction (6.7%), accommodation and food services (6.5%), manufacturing (5%), financial and insurance services (4.7%), transport, postal and warehousing (3.9%), information media and telecommunications (3.6%), arts and recreation services (3.3%), public administration and safety (3%), wholesale trade (1.7%), rental, hiring, and real estate services (1.6%), electricity, gas, water and waste services (1.4%), mining (1.2%), and other services (8.2%). About 40% reported having at least one chronic condition, with the most common being arthritis or rheumatism (17.6%) followed by asthma, emphysema, or chronic bronchitis (14.6%).

1. **Summary of Lockdown Dates and Restrictions During and Before Data Collection Periods**

Table S1. Summary of restrictions during the first wave of COVID-19 in Australia (early 2020)

|  | **VIC-Metro** | **VIC-Rural** | **NSW** | **QLD** | **TAS** | **WA** | **SA** | **NT** | **ACT** |
| --- | --- | --- | --- | --- | --- | --- | --- | --- | --- |
| *First reported case* | 25 Jan 2020 | | 25 Jan 2020 | 29 Jan 2020 | 2 Mar 2020 | 21 Feb 2020 | 1 Feb 2020 | 4 Mar 2020 | 12 Mar 2020 |
| *National lockdown* | 23 Mar 2020 | | 23 Mar 2020 | 23 Mar 2020 | 23 Mar 2020 | 23 Mar 2020 | 23 Mar 2020 | 23 Mar 2020 | 23 Mar 2020 |
| *Start of easing hard lockdown** | 11 May 2020  (7 weeks)  - up to 10 outdoors  - up to 5 for visiting at someone’s home  - up to 10 for weddings  - up to 20 for funerals held indoors and 30 for outdoors  - resumption of some outdoor recreational activities | | 1 May 2020  (6 weeks)  - up to two adults and dependent children to visit another household | 15 May 2020  (8 weeks)  - Stage 1 reopening:  - up to 10 in a public space  - recreational travel of a radius of up to 150km from the person’s home for day trips  - re-opening of libraries, playground equipment, skate parks and outdoor gyms, with up to 10 people at a time | 11 May 2020  (7 weeks)  - 20 people to attend funerals  - people to visit those living in residential aged care once a week  - TasTAFE campuses and training facilities to open for small groups of students | 27 April 2020  (5 weeks)  - Stage 1 reopening  - up to 10 for indoor and outdoor non-work gatherings | 11 May 2020  (7 weeks)  - Stage 1 reopening | 1 May 2020  (6 weeks)  - Stage 1 reopening Stage one adjustments for simple and safe outdoor activities where physical distancing can be maintained at all times. Including:  Personal gatherings including outdoor weddings and funerals.  Playgrounds, parks and campgrounds outside biosecurity areas.  Public swimming pools, lagoons and water parks.  Outdoor sports where physical distancing can be maintained including golf and tennis as well as training outdoors.  Go fishing, boating, and sailing with other people, real estate open house inspections and auctions, and gatherings in homes. | 15 May 2020  (8 weeks)  - cafés and restaurants to seat up to ten people |
| *Further easing of restriction* | 26 May 2020  - phased return to the classroom | | 11 May 2020  - return to face-to-face teaching  - increased retail activity  15 May 2020  - up to 10 for outdoor gatherings  - up to 10 for cafés and restaurants  - up to 5 visitors to a household  1 June 2020  - travel to regional NSW  - up to 50 for places of worship funeral  - camping grounds and caravan parks to open  1 July  - gyms and fitness studios to reopen (up to 10 per class and 100 people in an indoor venue)  - children’s sport and community sports competition for people aged up to 18 years to resume  - up to 10 for tattoo and massage parlours | 12 June 2020  - Stage 2 reopening  10 July  - borders open, except for VIC | 17 June 2020  - 20 visitors to a home in addition to household members  - groups of 20 to participate in social sports activities and  - 20 attendees per pool  13 July 2020  - Stage 3 reopening  24 July 2020  - borders reopen | 18 May 2020  - Stage 2 reopening  - up to 20 for indoor and outdoor non-work gatherings  - people encouraged to return to work, unless they are unwell or vulnerable  - up to 20 for cafés and restaurants  6 June 2020  - Stage 3 – up to 100 for  non-work indoor and outdoor  - up to 300 per venue  - food businesses and licensed premises to operate with seated service  - alcohol to be served without a meal at licensed premises  27 June 2020  - Stage 4 reopening | 15 May 2020  - opening of campsites and national parks with amended services to ensure social distancing  1 June 2020  - Stage 2 reopening: up to 80 for pubs, gyms, cinemas, places of worship, beauty salons and other sites  29 June 2020  - Step 3 reopening | 15 May 2020  - Stage 2 Reopening Stage two activities (safer indoor activities for less than two hours), such as:  Shopping centre food courts.  Restaurants, cafes, and bars for the consumption of food – excluding gaming areas.  Organised outdoor training activities for sport teams without physical contact.  Beauty therapy salons for non-facial services such as nails, massage and tanning. | 18 May 2020  - staged return to on-campus schooling  29 May 2020  - up to 50 people for funeral  - up to 20 for cafés, bars, restaurants and clubs  - beauty therapy  businesses to reopen  19 June 2020  - cafés, restaurants, bars and other licenced venues to seat up to 100  - contact sports to start full training from 19 June to start their transition back to competitions from 10 July. |

* Defined as the day any form of restriction was eased.

Table S2. Summary of lockdown and restrictions during our Study 2 survey period: 21 October – 10 November 2020, including the easing of restrictions from Melbourne/Victoria’s second wave of COVID-19 infections (July – November 2020)*.

|  | **VIC-Metro** | **VIC-Rural** | **NSW** | **QLD** | **TAS** | **WA** | **SA** | **NT** | **ACT** |
| --- | --- | --- | --- | --- | --- | --- | --- | --- | --- |
| *Face masks* | Mandatory | Mandatory | Not mandatory but strongly recommended. | Not mandatory but strongly recommended | Not mandatory but strongly recommended | Not mandatory but strongly recommended | Not mandatory but strongly recommended | Not mandatory but strongly recommended | Not mandatory but strongly recommended |
| *Curfew* | Not in place | Not in place | Not in place | Not in place | Not in place | Not in place | Not in place | Not in place | Not in place |
| *House visitors* | From 18 Oct:  1 nominated visitor if living alone/single parent (children >18)  Respite care for people with complex needs allowed  From 27 Oct:  Up to 2 from the same household per day, 25km rule applies | Up to 2 from different households per day | From 16 Oct:  up to 50 at a time, and no more than 30 strongly recommended if residence has no outdoor area | Up to 50 | Up to 40 | No limit, 2 sq m rule applies | Up to 10, 4 sq m rule applies | No limit, 1.5 sq m rule applies | No limit |
| *Outdoor gathering* | From 18 Oct:  Up to 10 from a maximum of 2 households  From 27 Oct:  Up to 10 from any number of households | Up to 70 | From 23 Oct:  Up to 30 (previously 20) | Up to 50 (does not apply to businesses operating with a COVID-safe plan | Up to 1,000, 2 sq m rule applies | No limit, 2 sq m rule applies | Up to 50, 4 sq m rule applies | No limit, 1.5 sq m rule applies Gatherings of >100 will require the completion of a COVID-safe plan. | Up to 500, 2 sq m rule applies |
| *Hospitality venues (restaurants, cafes, pubs)* | From 18 Oct:  Take-away and delivery only  From 27 Oct:  Up to 10 per table and group limit of 10, maximum of 20 for indoor and 50 for outdoor venues. | Up to 10 per indoor space with a maximum of 40 for indoor venues.  2 and 4 sq m rules apply.  Take-away only for people from metropolitan Melbourne | From 16 October:  2 sq m rule for outdoor areas (previously 4 sq m rule)  Functions of up to 300 people per venue, subject to a COVID-safe plan  From 23 October:  Up to 30 per booking and 30 per table (previously 10) | No limit, 2 sq m rule and COVID-safe plan applies | Up to 250 for indoor and 1,000 for outdoor venues, 2 sq m rule applies | No limit, 2 sq m rule and COVID-safe plan applies | Up to 100 per venue, and up to 10 per booking, 4 sq m rule applies, seated dining only | No restrictions, COVID-safe plan applies | Up to 500 per venue, 4 sq m rule applies |
| *Intra-state travel* | From 18 Oct:  25 km rule applies, (previously 5 km) Travel to regional Victoria only for permitted purposes, even if within 25 kms  From 27 Oct:  Travel to regional Victoria only for permitted purposes, even if within 25 kms  From 8 November:  Allowed | No information | No restrictions | No restrictions | No restrictions | No restrictions | No restrictions | No restrictions | No restrictions |
| *Inter-state travel* | From 18 Oct:  Not allowed, stay local, 25 km rule applies  From 27 Oct:  Not allowed, stay local, 25 km rule applies | From 18 Oct:  Not allowed, stay local, 25 km rule applies | Border to VIC closed (re-opened 23 November). | Open to all states except VIC  QLD Border Declaration Pass required if have been in a hotspot in the last 14 days, or overseas and did not fly into QLD when arrived in Australia. | Open to all states except VIC  Registration via the Tas e-Travel system required at least three days before entering TAS | Open to all states, except VIC  G2G PASS registration and declaration process required | Open to all states, except VIC  A cross-border travel registration required | Open to all states, except VIC  A border entry form and declaration required up to 72 hours before arrival. | Open to all states, except VIC  An online declaration required |
| *Wedding or funeral attendance*  *Note: Would only affect a person who had to cancel a wedding, or missed a wedding or funeral* | From 18 Oct:  **Weddings**:  In outdoor public spaces only with up to 5 people (including the couple and two witnesses but not including the celebrant)  **Funerals**:  Up to 10 people (not including people conducting the funeral)  From 27 Oct:  **Weddings:**  Up to 10 people outdoors (including the couple and two witnesses but not including the celebrant and 1 photographer)  **Funerals:**  Up to 20 people both indoors and outdoors (not including people conducting the funeral)  From 8 November: Up to 50 people outdoors (not including people conducting the funeral) | No Info | From 23 October:  Up to 300 for weddings and 100 for funerals, 4 and 2 sq m rule apply  Up to 20 people in the wedding party can dance on the dance floor, but only for members of the wedding party.  A record of names and contact details of each guest must be kept. | Up to 200 for weddings and funerals at professional venues.  Up to 30 for private weddings, including the wedding party and celebrant if there is no COCIV-safe plan.  A record of names and contact details of each guest must be kept for 56 days. | Up to 250 for indoor, and up to 1,000 for outdoor spaces, 2 sq m rule applies | No limit, 2 sq m rule applies | Up to 150 for weddings and 50 for funerals, 4 sq m rule apply for both | No limit, but a COVID-safe plan required for more than 100 people | Up to 500 for both weddings and funerals, 4 sq m rule apply for both |
| *Place of worship*  *Note: Would only affect a person who wished to attend a religious service* | From 18 Oct:  Places of worship closed  Up to 5 people, plus 1 faith leader for outdoor gatherings (not ceremonies), no sharing of food, drink or other items by participants  From 27 Oct:  Up to 10 plus 1 faith leader for indoor ceremonies, with cleaning requirements between services  Up to 20, plus 1 faith leader for outdoor religious gatherings, no sharing of food, drink or other items by participants. | Up to 20 people plus 1 faith leader for outdoor religious gatherings (previously 10), no sharing of food, drink, crockery, utensils, vessels or other equipment by participants | From 23 October:  Up to 300, 4 sq m rule applies, even if men and women are in separate areas. Reconsider activities that might spread the virus – singing and passing collection baskets. The use of face masks is strongly recommended for all services. | Up to 50, 4 sq m rule applies (2 sq m rule for venues less than 200 sq m) | Up to 250, 2 sq m rule applies | No limit, 2 sq m rule applies | Up to 100, 4 sq m rule applies | No limit, 1.5 sq m rule applies | Up to 25, excluding those conducting the service |
| *Schools* | From 12 Oct:  Staged return to onsite learning.  From 27 Oct:  Open | No Info (same as Metro Melb) | From 5 Oct: Resumes with strict COVID-safe plan  High school formals permitted from November 12. | Open | Open | Open | Open | Open | Open |
| *Salons, spas and other beauty services* | From 18 Oct:  Beauty/personal care services open, face mask mandatory for the duration of service | No Info (same as Metro Melb) | Open | Open | Open | Open | Open | Open | Open |
| *Cinemas, entertainment venues, museums, libraries and open houses* | From 18 Oct:  All venues closed  From 27 Oct:  Outdoor venues and spaces open with density quotients, patron caps and COVID-safe plans  From 8 November: Electronic gaming allowed subject to patron and time limits | Up to 20 for indoor libraries and toy libraries, 4 sq m rule applies | Open, 4 sq m rule applies, and a Covid-19 safety plan.  Up to 50% capacity and allocated seats for large venues | No limit, with a COVID-safe plan.  Face masks mandatory | Up to 250 for indoor and 1,000 for outdoor facilities, 2 sq m rule applies permitting | No limit, 2 sq m rule applies  Up to 50% capacity cap on major sport and entertainment venues.  Large scale, multi-stage music festivals prohibited | No limit, 4 sq m rule applies | No limit | Up to 50% capacity for each venue, allocated seats, 4 sq m rule applies |
| *Gym and exercise activities* | From 18 Oct:  **Outdoor recreation:**  25 km rule applies, can use outdoor sport and recreation facilities  Up to 2 people per trainer for outdoor personal training  Outdoor pools open, indoor swimming pools open for one-on-one hydrotherapy sessions with a health professional where clinically indicated  No group sessions  **Exercise:**  Up to 10 from 2 households for outdoor exercise  From 27 Oct:  **Sport and physical recreation for adults:**  Minimum number required to play game for outdoor non-contact sports, Up to 10 for outdoor fitness and fitness classes (excluding the trainer).    From 8 November:  Up to 10 people in a space and 20 in a venue, subject to density limits  **Indoor pools (including swimming classes):**  Open for one-on-one hydrotherapy sessions with a health professional, carer or support person  No group sessions  From 8 November:  Up to 20 per venue  **Outdoor swimming pools:**  Up to 50 or density limits, other than for exclusive use by a single school at any one time for education purposes or community sport | Up to 20 in the pool or 4 sq m rule (whichever is smaller)  One parent/guardian/ carer per child is permitted for supervision  Swimming classes can resume | Up to 20 per class, 4 sq m rule applies, excluding staff  Up to 500 for community sporting competitions and training, 4 sq m rule applies, excluding staff | Up to 50 indoor (2 or 4 sq m rule applies depending on size of venue)  No limit for outdoor non-contact sport and group training and boot camps, 4 sq m rule applies | Up to 250 for indoor venues, 2 sq m rule applies Up to 1,000 for outdoor gathering  Full contact training and full competition sport (contact and non-contact) is allowed, as is sharing equipment, change rooms and other facilities | No limits, 2 sq m rule applies Gyms can operate unstaffed but must undergo regular cleaning. Contact sport and training can also recommence, and playgrounds, outdoor gym equipment and skate parks can be used. | No limits for gyms, recreation centres, trampoline and play cafes, as well as outdoor fitness activities, such as boot camps and personal training sessions, 4 sq m rule applies Indoor play centres, amusement parks and arcades remain closed, as well as community or club sports fixtures and trainings, whether indoors or outdoors  Swimming only for fitness or rehabilitation | No limits for gyms, fitness studios and indoor training activities such as Cross Fit, as well as team sports such as football, basketball, soccer and netball. | Up to 100 for indoor gyms and fitness centres, 4 sq m rule applies. |
| *Restrictions prior to our data collection following second wave of infections in Victoria* | 2 Aug 2020 – 13 Sept 2020 VIC a State of Disaster. **Stage 4 restrictions for Metro Melbourne**:  - curfew (8pm-5am)  - compulsory face masks  - four essential reasons for leaving the house  - exercise: 2 hours max, with 1 other person only  - shopping: 1 person per household  - 5km rule  - Remote learning | **Stage 3 restrictions from 5 August 2020 for Regional VIC**  - Four reasons to be out  - Compulsory face masks  - Cafes and restaurants takeaway only  - Nonessential shops to close  - No community sport  - Remote learning |  |  |  |  |  |  |  |

***** During our data collection period (21 October to 10 November 2020), Public Health Orders remained within the remit of each state and territory; therefore, the type and duration of restrictions varied widely across the country, as summarised in this document.

**Key dates:**

13 Sept 2020 – ‘Roadmap’ for reducing restrictions in Victoria commences (see [Victoria’s roadmap for reopening – How we live in Metropolitan Melbourne (amaze.org.au)](https://www.amaze.org.au/wp-content/uploads/2020/09/Victoria-roadmap-Melbourne-Metro_0.pdf) for details)

19 Oct 2020 – Restrictions are significantly eased in Victoria, with further easing on 27 October as cases continue to be less than 5

26 Oct 2020 – VIC records zero new cases and deaths for the first time since 9 June

**Sources:**

NSW: <https://www.health.nsw.gov.au/news/Pages/2020-nsw-health.aspx>

VIC: <https://www.theguardian.com/australia-news/datablog/ng-interactive/2020/oct/20/coronavirus-australia-map-cases-covid-19-tracking-stats-live-data-update-by-state-suburb-postcode-how-many-new-active-case-numbers-today-statistics-corona-deaths-death-toll>

QLD: <https://www.theguardian.com/australia-news/datablog/ng-interactive/2020/oct/20/coronavirus-australia-map-cases-covid-19-tracking-stats-live-data-update-by-state-suburb-postcode-how-many-new-active-case-numbers-today-statistics-corona-deaths-death-toll>

SA: <https://www.covid-19.sa.gov.au/home/dashboard/dashboard-table-data#covid-19-daily>

WA: <https://covidlive.com.au/wa>

ACT: <https://app.powerbi.com/view?r=eyJrIjoiZTY4NTI1NzQtYTBhYy00ZTY4LTk3NmQtYjBjNzdiOGMzZjM3IiwidCI6ImI0NmMxOTA4LTAzMzQtNDIzNi1iOTc4LTU4NWVlODhlNDE5OSJ9>

NT: <https://coronavirus.nt.gov.au/>;

<https://newsroom.nt.gov.au/mediaRelease/33205>

1. **Newly Developed COVID-19 Measures**

**COVID-19 Beliefs**

Rate the extent to which you agree or disagree with these statements.

Response scale: 1=strongly disagree, 2=disagree, 3=neither agree nor disagree, 4=agree, 5=strongly agree.

Items:

1. Social distancing is effective in slowing the spread of COVID-19
2. Social distancing is destroying our economy
3. Social distancing for children is unnecessary and adds burden on parents
4. If we didn’t practice social distancing, the curve will get steeper and the number of COVID-19 cases and deaths will increase
5. A flatter curve means less burden on the healthcare system
6. The Australian Government should test, track and trace every potential case of COVID-19
7. We should rely on people getting COVID-19 in order to build up (herd) immunity

Scoring:

Response Efficacy Beliefs: Items 1, 4, 5, 6; Perceived Barriers Beliefs: Items 2, 3, 7

**COVID-19 Impact Index**

Response scale: -10 to +10

What impact has COVID-19 had on your:

1. Time availability?

(*bored with too much time on my hands* to *overwhelmed with competing tasks*)

1. Job security?

(*worse off* to *better off*)

1. Financial situation?

(*worse off* to *better off*)

1. Household/family responsibilities (e.g., daily chores, parenting, schooling children)? (*much less responsibilities* to *much more responsibilities*)
2. Extended family responsibilities (caring for people not living in your household e.g., shopping, medical care, emotional support)?

(*much less responsibilities* to *much more responsibilities*)

1. Nutrition/diet?

(*worse off* to *better off*)

1. Amount of physical activity (intentional and/or incidental)?

(*much less active* to *much more active*)

1. Quality of sleep?

(*worse off* to *better off*)

1. Family relationships?

(*worse off* to *better off*)

1. Mental health?

(*worse off* to *better off*)

1. Physical health?

(*worse off* to *better off*)

1. Quality of social connections (including via digital means)?

(*worse off* to *better off*)

1. Alcohol consumption?

(*much less* to *much more*)

1. Substance use?

(*much less use* to *much more use*)

1. Living situation (e.g., living space)?

(*much less comfortable* to *much more comfortable*)

1. How lonely you feel?

(*much less lonely* to *much more lonely*)

1. Hobbies/leisure activities?

(*much less time spent on hobbies/leisure activities* to *much more time sent on hobbies/leisure activities*)

1. Daily routine?

(*much less structured* to *much more structured*)

1. Work/study productivity?

(*much less productive* to *much more productive*)

Scoring: details and syntax are available upon request from the corresponding author.

**Multidimensional COVID-19 Worry Scale**

To what extent do the following statements describe how you feel NOW:

*1=never; 4=always*

1. I am nervous when I think about the pandemic
2. I am calm and relaxed when I think about the pandemic*
3. I am worried about my health due to COVID-19
4. I am worried about the health of my family members due to COVID-19
5. I am stressed about leaving my house
6. I am worried about someone I love dying due to COVID-19 related reasons
7. I am worried about returning to face-to-face interactions
8. I am stressed about taking public transport
9. I am concerned about the possibility of another wave of COVID-19 in the city/region where I live, or where my friends/family live
10. I am concerned about the possibility of another lockdown in the city/region where I live, or where my friends/family live
11. I am worried about losing money
12. I am worried about becoming unemployed
13. I am worried about global economic recession
14. I am worried about political systems failing
15. I am worried about my ﬁnancial situation
16. I am worried about the Australian economy
17. I am worried about society and social liberties degrading
18. I am worried about grocery stores running out of food and/or other supplies
19. I am worried about pharmacies running out of medicines/essential health supplies
20. I am worried about our healthcare system being overloaded
21. I am worried about the healthcare system not being able to protect me or my loved ones

**reverse scored*

Scoring:

Personal/Family Concerns: Items 1, 2, 3, 4, 5, 6, 7, 8, 9, 10

Personal Financial Concerns: Items 11, 12, 15

Economy/Liberties Concerns: Items 13, 14, 16, 17

Infrastructure/Supplies Concerns: Items 18, 19, 20, 21

**Social Support**

Items:

1. How many people were so close to you that you could count on them if you had great personal problems?

(1=none; 2=1-2; 3=3-5; 4=5+)

1. How much interest did people show in what you did?

(1=none; 2=little; 3=uncertain; 4=some; 5=a lot)

1. How easy was it to get practical help from neighbours if you needed it?

(1=very difficult; 2=difficult; 3=possible; 4=easy; 5=very easy)

1. How many people were so close to you that you could count on them if you had non-COVID related physical health problems?

(1=none; 2=1-2; 3=3-5; 4=5+)

1. How many people were so close to you that you could count on them if you had COVID-related physical health problems?

(1=none; 2=1-2; 3=3-5; 4=5+)

1. How many people were so close to you that you could count on them if you had COVID-related mental health problems?

(1=none; 2=1-2; 3=3-5; 4=5+)

Items 1 to 3 are from the Oslo Social Support Scale (Dalgard, 1996). The scale was extended to include items 4 to 6 which are modifications of item 1 with specific reference to COVID-19 health problems. The modified scale remained uni-dimensional, with all items converging onto a single factor.

1. **Descriptive Statistics and Correlations**

Table S3

*Pearson correlations between Character Growth Awareness Scale items for Study 1 (N=417) and Study 2 (N=1898)*

|  | 1 | 2 | 3 | 4 | 5 |
| --- | --- | --- | --- | --- | --- |
| 1. Going through the lockdown made me realise that I'm stronger than I thought I was | 1 | .52*** | .64*** | .63*** | .61*** |
| 2. The lockdown made me reflect on the type of person I am | .58*** | 1 | .64*** | .68*** | .61*** |
| 3. I have a better understanding of how resilient I am as a result of the pandemic experience | .62*** | .50*** | 1 | .73*** | .72*** |
| 4. Experiencing the pandemic has built my strength of character | .66*** | .59*** | .71*** | 1 | .82*** |
| 5. I have a better understanding of how to adapt to challenging situations as a result of the pandemic experience | .63*** | .54*** | .73*** | .80*** | 1 |

*Note.* Study 1 correlations are reported above the diagonal and Study 2 below the diagonal.

**** p*<.001

Table S4

*Descriptive Statistics and Reliability Estimates (Cronbach’s alpha) for all Measures in Study 1*

|  | Mean | SD | *α* |
| --- | --- | --- | --- |
| Dependent Variables |  |  |  |
| Mental Well-being | 46.46 | 9.91 | .93 |
| Mental Well-being Recovery | 3.71 | 7.86 | - |
| Demographics |  |  |  |
| Age | 34.38 | 12.92 | - |
| Education Level | 5.29 | 1.54 | - |
| Annual Income | 3.30 | 1.89 | - |
| Household Size | 3.02 | 1.34 | - |
| Physical Health Symptoms | 1.80 | .99 | .83 |
| Financial Comfort | 55.95 | 25.96 | - |
| Social Support | 2.69 | .66 | .82 |
| Social Desirability | 6.76 | 2.96 | .72 |
| Personality |  |  |  |
| Extraversion | 10.22 | 3.73 | .80 |
| Agreeableness | 14.99 | 3.38 | .83 |
| Conscientiousness | 14.01 | 3.05 | .67 |
| Neuroticism | 11.24 | 3.43 | .76 |
| Intellect/Openness | 14.59 | 3.35 | .75 |
| COVID-19 Measures |  |  |  |
| Positive Impact | 15.85 | 16.15 | .87 |
| Negative Impact | 30.07 | 21.28 | .85 |
| Response Efficacy Beliefs | 4.45 | .54 | .65 |
| Perceived Barrier Beliefs | 2.16 | .73 | .53 |
| CF2 Constructs |  |  |  |
| Lack of Impulse/Self-Control^a^ | - | - | n/a |
| Resilience/Adaptability^a^ | - | - | .86 |
| COVID-19 CGA | 3.78 | 1.39 | .86 |

Note. *α* = Cronbach’s alpha. MWB = mental well-being.

^a^ Means, standard deviations and reliability estimates for the variables included in these composites (Bartlett scores) are as follows:

For the Lack of Impulse/Self-Control composite: Self-Control: M = 41.12 (SD = 8.87), α = .86; Impulsivity (Emotion-Based): M = 2.01 (SD = .62), *α* = .86; Impulsivity (Sensation-Seeking): M = 2.29 (SD = .72), *α* = .72; Impulsivity (Conscientiousness Deficits): M = 1.79 (SD = .45), *α* = .81.

For the Resilience/Adaptability composite: Resilience: M = 26.09 (SD = 6.28), *α* = .88; Adaptability (Handling Crises): M = 21.74 (SD = 4.10), *α* = .88, Adaptability (Tolerance for Uncertainty): M = 29.63
 (SD = 5.16), *α* = .82.

**p*<.05; ***p*<.01.

Table S5

*Descriptive Statistics and Reliability Estimates (Cronbach’s alpha) for all Measures in Study 2*

| Variable | Mean (SD) | SD | *α* |
| --- | --- | --- | --- |
| Dependent Variables |  |  |  |
| Kessler-10 | 19.43 | 9.65 | .96 |
| GAD-7 | 4.77 | 5.44 | .95 |
| Demographics |  |  |  |
| Age | 47.43 | 17.81 | - |
| Education | 3.53 | 1.64 | - |
| Chronic Comorbidities | .63 | 0.95 | - |
| Financial Comfort | 6.54 | 2.85 | - |
| COVID-19 Measures |  |  |  |
| Positive COVID-19 Impact | 3.51 | 3.98 | .91 |
| Negative COVID-19 Impact | 6.96 | 6.28 | .93 |
| Response Efficacy Beliefs | 4.07 | 0.82 | .84 |
| Perceived Barriers Beliefs | 2.69 | 0.93 | .68 |
| Personal/Family Concerns | 2.38 | 0.69 | .90 |
| Supplies/Infrastructure Concerns | 2.06 | 0.83 | .90 |
| Economy/Liberties Concerns | 2.49 | 0.78 | .83 |
| Personal Financial Concerns | 2.20 | 0.91 | .84 |
| Intolerance of Uncertainty | 29.68 | 10.78 | .93 |
| COVID-19 Character Growth Awareness | 4.81 | 1.37 | .90 |

**Relationships between demographics and the dependent variables: Independent samples t-tests**

Table S4 presents correlations between all continuous variables in Study 1. To examine the relationships between categorical variables (sex and state) and main variables of interest, independent samples t-tests were run. There were no significant differences between males and females on Mental Well-being and Mental Well-being Recovery, *t*(412) = .40, *p* = .29 and *t*(412) = -1.39, *p* = .17. Females scored significantly higher on COVID-19 CGA (M = 3.93, SD = 1.27), than males (M = 3.61, SD = 1.47); however, these differences were weak, *t*(412) = -2.40, *p* = .02.

There were no statistically significant differences between participants living in NSW (n=131) and other states (n=286) on Mental Well-being, *t*(415) = -.18, *p* = .86; and Mental Well-being Recovery, *t*(415) = -.563, *p* = .57. However, participants residing in NSW (M=4.05, SD=1.27) reported higher levels of COVID-19 CGA compared with other states (M=3.65, SD=1.42), *t*(415) = -.2.80, *p* = .001.

With two notable differences, similar results were received for participants living in Victoria (n=119) compared to the other states (n=298). There were no statistically significant differences between participants living in Victoria and other states on Mental Well-being *t*(415) = .85, *p* = .40. However, participants living in Victoria reported significantly smaller degrees of recovery in their mental well-being (M=2.41, SD=6.80) compared to people living in all other states (M=4.2, SD=8.2), *t*(415) = 2.14, *p* = .03). This finding is expected, and supports the authenticity of the testing period, given that at the time of testing, Victoria was the only state facing a second lockdown. Lastly, there were no significant differences in COVID-19 CGA reported by participants residing in Victoria compared with other states, *t*(415) = 8.5, *p* = .40.

Table S5 presents correlations between all continuous measures in Study 2. Independent samples t-tests were run to examine the relationships between categorical and dependent variables. Females scored significantly higher on psychological distress (M = 19.96, SD = 9.69) than males (M = 18.86, SD = 9.59), *t*(1896) = -2.49, *p* = .013. Similarly, females scored significantly higher on generalised anxiety (M = 5.08, SD = 5.56) than males (M = 4.27, SD = 5.29), *t*(1896), *p =* .009.

There were no significant differences between those residing in Victoria (n=954) and those in all other states (n=944) on psychological distress, *t*(1896) = -.35, *p* = .73; or anxiety, *t*(1896) = -1.15, *p* = .25. Participants living in NSW did, however, score significantly lower on psychological distress (M = 18.41, SD = 9.60) compared to those in other states (M = 19.62, SD = 9.65), *t*(1896) = 1.96, *p* = .05. Participants living in NSW also scored significantly lower on generalised anxiety (M = 4.19, SD = 5.38) than those in other states (M = 4.87, SD = 5.45), *t*(1896) = 1.98, *p* = .05.

Australian citizens/permanent residents scored significantly lower on psychological distress (M = 19.28, SD = 9.63) than those without citizenship or residency (M = 22.38, SD = 9.65), *t*(1896) = 2.97, *p* = .003. Similarly, Australian citizens/permanent residents were significantly lower on generalised anxiety (M = 4.68, SD = 5.42) than others (M = 6.46, SD = 5.57), *t*(1896) = 3.02, *p* = .003.

Aboriginal and Torres Strait Islanders scored significantly higher on psychological distress (M = 25.18, SD = 9.66) than non-Indigenous (M = 19.20, SD = 9.58), *t*(1896) = -5.26, *p* < .001. They also scored significantly higher on generalised anxiety (M = 7.85, SD = 4.81) than non-Indigenous (M = 4.64, SD = 5.43), *t*(1896) = -5.01, *p* < .001.

Table S6

*Correlations Between All Continuous Measures in Study 1*

|  | 1 | 2 | 3 | 4 | 5 | 6 | 7 | 8 | 9 | 10 | 11 | 12 | 13 | 14 | 15 | 16 | 17 | 18 | 19 | 20 | 21 |
| --- | --- | --- | --- | --- | --- | --- | --- | --- | --- | --- | --- | --- | --- | --- | --- | --- | --- | --- | --- | --- | --- |
| 1 MWB |  |  |  |  |  |  |  |  |  |  |  |  |  |  |  |  |  |  |  |  |  |
| 2 MWB Recovery | .32** |  |  |  |  |  |  |  |  |  |  |  |  |  |  |  |  |  |  |  |  |
| 3 Age | .09 | -.12* |  |  |  |  |  |  |  |  |  |  |  |  |  |  |  |  |  |  |  |
| 4 Education | .07 | -.07 | .11* |  |  |  |  |  |  |  |  |  |  |  |  |  |  |  |  |  |  |
| 5 Income | .12* | -.06 | .31** | .43** |  |  |  |  |  |  |  |  |  |  |  |  |  |  |  |  |  |
| 6 Household Size | .05 | .12* | -.23** | -.11** | -.11* |  |  |  |  |  |  |  |  |  |  |  |  |  |  |  |  |
| 7 Physical Health | -.33** | .06 | -.05 | -.03 | .02 | -.01 |  |  |  |  |  |  |  |  |  |  |  |  |  |  |  |
| 8 Financial Comfort | .31** | .05 | .03 | .10* | .23** | -.08 | -.13** |  |  |  |  |  |  |  |  |  |  |  |  |  |  |
| 9 Social Support | .42** | .01 | .01 | .09 | .06 | .08 | -.10* | .23** |  |  |  |  |  |  |  |  |  |  |  |  |  |
| 10 Social Desirability | .31** | .01 | .12* | -.05 | -.01 | .03 | -.23** | .09 | .13** |  |  |  |  |  |  |  |  |  |  |  |  |
| 11 Extraversion | .33** | .17** | -.03 | .04 | .13* | .08 | .04 | .11* | .30** | .05 |  |  |  |  |  |  |  |  |  |  |  |
| 12 Agreeableness | .28** | .03 | .03 | -.01 | -.01 | -.03 | .03 | .11* | .29** | .22** | .36** |  |  |  |  |  |  |  |  |  |  |
| 13 Conscientiousness | .28** | .09 | .21** | .04 | .11* | -.05 | -.19** | .08 | .17** | .30** | -.01 | .19** |  |  |  |  |  |  |  |  |  |
| 14 Neuroticism | -.53** | .05 | -.16** | -.08 | -.16* | .01 | .42** | -.20** | -.30** | -.39** | -.14** | -.01 | -.29** |  |  |  |  |  |  |  |  |
| 15 Intellect | .22** | .02 | -.04 | .07 | .03 | -.04 | .02 | .09 | .15** | .08 | .25** | .27** | .08 | -.05 |  |  |  |  |  |  |  |
| 16 Positive COVID Impact | .21** | .14** | -.14** | .05 | .09 | .07 | .08 | .09 | .09 | .07 | .09 | .02 | .02 | -.06 | .01 |  |  |  |  |  |  |
| 17 Negative COVID Impact | -.30** | .08 | -.20** | -.07 | -.09 | .02 | .36** | -.33** | -.18** | -.11* | .06 | .05 | -.13** | .28** | .04 | -.08 |  |  |  |  |  |
| 18 Response Efficacy | .05 | .01 | -.11* | .04 | .07 | .02 | -.03 | .11* | .14** | .01 | .10* | .25** | .09 | -.09 | .10 | .02 | .12* |  |  |  |  |
| 19 Barriers Beliefs | .00 | .00 | .25** | .03 | .10 | .09 | .05 | -.01 | .00 | .00 | .07 | -.08 | -.02 | .07 | -.06 | .01 | -.10* | -.41** |  |  |  |
| 20 Impulsivity/Lack of Self-Control | -.36** | .03 | -.26** | -.10* | -.05 | .15** | .26** | -.13* | -.22** | -.39** | .09 | -.21** | -.58** | .41** | -.06 | .00 | .20** | -.12* | .09 |  |  |
| 21 Resilience/ Adaptability | .53** | .06 | .12* | .11* | .21** | .02 | -.16** | .18** | .32** | .30** | .34** | .21** | .28** | -.49** | .32** | .14** | -.12* | .15** | .00 | -.19** |  |
| 22 COVID-19 CGA | .33** | .23** | -.04 | .00 | .10* | .01 | .05 | .10* | .20** | .12* | .18** | .17** | .11* | -.10 | .05 | .36** | .02 | .05 | .05 | -.06 | .29** |

*Note.* **p* < .05; ***p* < .01.

Table S7

*Correlations Between all Continuous Measures in Study 2*

|  | 1 | 2 | 3 | 4 | 5 | 6 | 7 | 8 | 9 | 10 | 11 | 12 | 13 | 14 | 15 | 16 |
| --- | --- | --- | --- | --- | --- | --- | --- | --- | --- | --- | --- | --- | --- | --- | --- | --- |
| 1 Kessler-10 |  |  |  |  |  |  |  |  |  |  |  |  |  |  |  |  |
| 2 GAD-7 | .89** |  |  |  |  |  |  |  |  |  |  |  |  |  |  |  |
| 3 Age | -.37** | -.35** |  |  |  |  |  |  |  |  |  |  |  |  |  |  |
| 4 Education | .03 | .02 | -.20** |  |  |  |  |  |  |  |  |  |  |  |  |  |
| 5 Comorbidities | .14** | .12** | .23** | -.12** |  |  |  |  |  |  |  |  |  |  |  |  |
| 6 Risk | .20** | .19** | -.13** | -.01 | .22** |  |  |  |  |  |  |  |  |  |  |  |
| 7 Financial Comfort | -.27** | -.26** | .06** | .14** | -.09** | -.00 |  |  |  |  |  |  |  |  |  |  |
| 8 Positive COVID-19 Impact | .11** | .10** | -.18** | .15** | .02 | .24** | .17** |  |  |  |  |  |  |  |  |  |
| 9 Negative COVID-19 Impact | .47** | .48** | -.25** | .07** | .08** | .06** | -.35** | -.03 |  |  |  |  |  |  |  |  |
| 10 Response Efficacy Beliefs | -.18** | -.15** | .29** | .01 | .04 | -.16** | .14** | .05* | -.08** |  |  |  |  |  |  |  |
| 11 Barriers Beliefs | .23** | .19** | -.14** | -.01 | -.00 | .11** | -.01 | .07** | .13** | -.37** |  |  |  |  |  |  |
| 12 Personal Family Concerns | .44** | .44** | -.17** | .05* | .14** | .04 | -.25** | .19** | .43** | .14** | -.00 |  |  |  |  |  |
| 13 Supplies/Infrastructure Concerns | .41** | .41** | -.23** | .00 | .10** | .06** | -.22** | .15** | .35** | -.03 | .15** | .69 |  |  |  |  |
| 14 Economy/Liberties Concerns | .27** | .29** | .00 | .02 | .08** | -.01 | -.22** | .13** | .38** | .03 | .17** | .54** | .56** |  |  |  |
| 15 Personal Financial Concerns | .46** | .46** | -.37** | .09** | -.02 | .02 | -.44** | .11** | .54** | -.07** | .13** | .56** | .53** | .53** |  |  |
| 16 Intolerance of Uncertainty | .59** | .56** | -.27** | .05* | .05* | .10** | -.16** | .14** | .32** | -.03 | .23** | .40** | .34** | .31** | .38** |  |
| 17 COVID-19 CGA | -.16** | -.14** | .05* | .08** | -.02 | -.03 | .24** | .27** | -.11** | .35** | -.06** | .09** | .05* | .07** | -.02 | -.02 |

*Note.* * *p* < .05; ** *p* < .01; *** *p* < .001.

1. **Regression Results**

Table S8

*Tolerance and Variance Inflation Factor Estimates for Regression Models in Study 1*

| Predictor | Tolerance | VIF |
| --- | --- | --- |
| Block 1 |  |  |
| Age | .68 | 1.46 |
| Sex | .79 | 1.26 |
| Victoria | .78 | 1.29 |
| NSW | .77 | 1.30 |
| Educational Attainment | .77 | 1.30 |
| Annual Income | .65 | 1.55 |
| Household Number | .87 | 1.15 |
| Physical Symptoms | .71 | 1.42 |
| Financial Comfort | .77 | 1.30 |
| Social Support | .72 | 1.39 |
| Social Desirability | .70 | 1.43 |
| Block 2 |  |  |
| Extraversion | .69 | 1.46 |
| Agreeableness | .64 | 1.55 |
| Conscientiousness | .61 | 1.64 |
| Neuroticism | .46 | 2.16 |
| Intellect | .78 | 1.28 |
| Block 3 |  |  |
| Positive COVID-19 Impact | .81 | 1.24 |
| Negative COVID-19 Impact | .69 | 1.46 |
| Block 4 |  |  |
| Response Efficacy Beliefs | .73 | 1.38 |
| Perceived Barriers Beliefs | .73 | 1.37 |
| Block 5 |  |  |
| Impulsivity/Lack of Self Control | .48 | 2.08 |
| Resilience/Adaptability | .54 | 1.86 |
| COVID-19 CGA | .76 | 1.32 |

Table S9

*Tolerance and Variance Inflation Factor Estimates for Regression Models in Study 2*

| Predictor | Tolerance | VIF |
| --- | --- | --- |
| Block 1 |  |  |
| Age | .59 | 1.70 |
| Sex | .97 | 1.04 |
| Victoria | .80 | 1.25 |
| NSW | .75 | 1.34 |
| Australian Citizenship/Residency | .94 | 1.07 |
| Indigenous Origin | .85 | 1.18 |
| Educational Attainment | .89 | 1.12 |
| Chronic Comorbidities | .83 | 1.21 |
| Health Risk Factors | .78 | 1.28 |
| Financial Comfort | .68 | 1.48 |
| Block 2 |  |  |
| Positive COVID-19 Impact | .80 | 1.25 |
| Negative COVID-19 Impact | .60 | 1.67 |
| Block 3 |  |  |
| Response Efficacy Beliefs | .65 | 1.55 |
| Perceived Barriers Beliefs | .76 | 1.32 |
| Block 4 |  |  |
| Personal/Family Concerns | .38 | 2.63 |
| Infrastructure/Supplies Concerns | .43 | 2.34 |
| Economy/Liberties Concerns | .52 | 1.91 |
| Personal Financial Concerns | .41 | 2.44 |
| Block 4 |  |  |
| Intolerance of Uncertainty (IU) | .65 | 1.53 |
| IU Square Term | .87 | 1.15 |
| Block 5 |  |  |
| COVID-19 CGA | .78 | 1.28 |

Table S10

*Regression Model Predicting Mental Well-Being Reported Post-Lockdown*

| Predictor | B | SE | *β* | sr^2^ | R^2^ | ΔR^2^ |
| --- | --- | --- | --- | --- | --- | --- |
| Block 1 |  |  |  |  | .34 | .34*** |
| Age | 0.03 | .03 | .04 | .00 |  |  |
| Gender | -0.42 | .84 | -.02 | .00 |  |  |
| Victoria | -0.59 | 1.01 | -.03 | .00 |  |  |
| NSW | -0.15 | .99 | -.01 | .00 |  |  |
| Educational Attainment | 0.04 | .30 | .01 | .00 |  |  |
| Annual Income | 0.28 | .26 | .05 | .00 |  |  |
| Household Number | 0.30 | .32 | .04 | .00 |  |  |
| Physical Symptoms | -2.24 | .44 | -.22*** | .04 |  |  |
| Financial Comfort | 0.07 | .02 | .18*** | .03 |  |  |
| Social Support | 4.92 | .65 | .33*** | .10 |  |  |
| Social Desirability | 0.65 | .15 | .20*** | .04 |  |  |
| Block 2 |  |  |  |  | .48 | .14*** |
| Extraversion | 0.39 | .11 | .15** | .02 |  |  |
| Agreeableness | 0.36 | .13 | .12** | .01 |  |  |
| Conscientiousness | 0.24 | .14 | .07 | .00 |  |  |
| Neuroticism | -0.98 | .14 | -.34*** | .06 |  |  |
| Intellect | 0.30 | .12 | .10* | .01 |  |  |
| Block 3 |  |  |  |  | .51 | .03*** |
| Positive COVID-19 Impact | 0.10 | .02 | .16*** | .02 |  |  |
| Negative COVID-19 Impact | -0.04 | .02 | -.09* | .00 |  |  |
| Block 4 |  |  |  |  | .51 | .01 |
| Response Efficacy Beliefs | -1.32 | .77 | -.07 | .00 |  |  |
| Perceived Barriers Beliefs | -0.03 | .58 | .00 | .00 |  |  |
| Block 5 |  |  |  |  | .57 | .06*** |
| Impulsivity/Lack of Self Control | -1.37 | .49 | -.17** | .01 |  |  |
| Resilience/Adaptability | 2.17 | .43 | .20*** | .02 |  |  |
| COVID-19 CGA | 1.55 | .39 | .16*** | .00 |  |  |

*Note. *p*<.05; ***p*<.01; ****p*<.001.

Table S11

*Regression Model Predicting Perceived Recovery in Mental Well-Being (from lockdown to post-lockdown)*

| Predictor | B | SE | *β* | sr^2^ | R2 | ΔR2 |
| --- | --- | --- | --- | --- | --- | --- |
| Block 1 |  |  |  |  | .05 | .05* |
| Age | -0.06 | .03 | -.10 | .01 |  |  |
| Gender | 0.86 | .80 | .05 | .00 |  |  |
| Victoria | -2.10 | .97 | -.12* | .01 |  |  |
| NSW | -0.75 | .94 | -.04 | .00 |  |  |
| Educational Attainment | -0.22 | .29 | -.04 | .00 |  |  |
| Annual Income | -0.06 | .25 | -.01 | .00 |  |  |
| Household Number | 0.56 | .30 | .10 | .01 |  |  |
| Physical Symptoms | 0.47 | .42 | .06 | .00 |  |  |
| Financial Comfort | 0.02 | .02 | .08 | .01 |  |  |
| Social Support | -0.22 | .62 | -.02 | .00 |  |  |
| Social Desirability | -0.06 | .03 | -.10 | .00 |  |  |
| Block 2 |  |  |  |  | .10 | .05** |
| Extraversion | 0.44 | .12 | .21*** | .03 |  |  |
| Agreeableness | -0.18 | .14 | -.08 | .00 |  |  |
| Conscientiousness | 0.43 | .14 | .17** | .02 |  |  |
| Neuroticism | 0.17 | .15 | .07 | .00 |  |  |
| Intellect | -0.01 | .12 | .00 | .00 |  |  |
| Block 3 |  |  |  |  | .12 | .02* |
| Positive COVID-19 Impact | 0.06 | .02 | .12* | .01 |  |  |
| Negative COVID-19 Impact | 0.03 | .02 | .09 | .01 |  |  |
| Block 4 |  |  |  |  | .12 | .00 |
| Response Efficacy Beliefs | -0.27 | .82 | -.02 | .00 |  |  |
| Perceived Barriers Beliefs | 0.01 | .61 | .00 | .00 |  |  |
| Block 5 |  |  |  |  | .15 | .03** |
| Impulsivity/Lack of Self Control | 0.22 | .54 | .02 | .00 |  |  |
| Resilience/Adaptability | 0.13 | .47 | -.03 | .00 |  |  |
| COVID-19 CGA | 1.58 | .43 | .20*** | .03 |  |  |

Table S12

*Regression Model Predicting Kessler-10*

| Predictor | B | SE | *β* | R^2^ | ΔR^2^ |
| --- | --- | --- | --- | --- | --- |
| Block 1 |  |  |  | .25 | .25 |
| Age | -.21 | .01 | -.38*** |  |  |
| Sex | 1.19 | .39 | .06** |  |  |
| Victoria | -.16 | .43 | -.01 |  |  |
| NSW | -1.22 | .59 | -.05* |  |  |
| Australian Citizenship/Residency | .73 | .93 | .02 |  |  |
| Indigenous Origin | .68 | 1.07 | .01 |  |  |
| Educational Attainment | .13 | .12 | .02 |  |  |
| Chronic Comorbidities | 1.84 | .22 | .18*** |  |  |
| Health Risk Factors | 2.15 | .42 | .11*** |  |  |
| Financial Comfort | -.77 | .07 | -.23*** |  |  |
| Block 2 |  |  |  | .36 | .10 |
| Positive COVID-19 Impact | .20 | .05 | .08*** |  |  |
| Negative COVID-19 Impact | .55 | .03 | .36*** |  |  |
| Block 3 |  |  |  | .37 | .02 |
| Response Efficacy Beliefs | -.01 | .25 | -.00 |  |  |
| Perceived Barriers Beliefs | 1.47 | .21 | .13*** |  |  |
| Block 4 |  |  |  | .43 | .06 |
| Personal/Family Concerns | 3.22 | .38 | .23*** |  |  |
| Infrastructure/Supplies Concerns | .54 | .31 | .05 |  |  |
| Economy/Liberties Concerns | -.69 | .30 | -.06* |  |  |
| Personal Financial Concerns | 1.13 | .29 | .11*** |  |  |
| Block 5 |  |  |  | .54 | .11 |
| Intolerance of Uncertainty (IU) | 3.34 | .19 | .35*** |  |  |
| IU Square Term | .65 | .13 | .09*** |  |  |
| Block 6 |  |  |  | .55 | .01 |
| COVID-19 CGA | -.74 | .12 | -.11*** |  |  |

Table S13

*Regression Model Predicting GAD-7*

| Predictor | B | SE | *β* | R^2^ | ΔR^2^ |
| --- | --- | --- | --- | --- | --- |
| Block 1 |  |  |  | .22 | .22 |
| Age | -.11 | .01 | -.35*** |  |  |
| Sex | .70 | .22 | .06** |  |  |
| Victoria | .14 | .25 | .01 |  |  |
| NSW | -.55 | .34 | -.04 |  |  |
| Australian Citizenship/Residency | .26 | .54 | .01 |  |  |
| Indigenous Origin | .40 | .62 | .01 |  |  |
| Educational Attainment | .04 | .07 | .01 |  |  |
| Chronic Comorbidities | .87 | .13 | .15*** |  |  |
| Health Risk Factors | 1.24 | .24 | .12*** |  |  |
| Financial Comfort | -.42 | .04 | -.22*** |  |  |
| Block 2 |  |  |  | .33 | .11 |
| Positive COVID-19 Impact | .11 | .03 | .08*** |  |  |
| Negative COVID-19 Impact | .33 | .02 | .37*** |  |  |
| Block 3 |  |  |  | .34 | .01 |
| Response Efficacy Beliefs | .03 | .14 | .00 |  |  |
| Perceived Barriers Beliefs | .58 | .12 | .10*** |  |  |
| Block 4 |  |  |  | .40 | .06 |
| Personal/Family Concerns | 1.77 | .22 | .23*** |  |  |
| Infrastructure/Supplies Concerns | .28 | .18 | .04 |  |  |
| Economy/Liberties Concerns | -.22 | .17 | -.03 |  |  |
| Personal Financial Concerns | .64 | .17 | .11*** |  |  |
| Block 4 |  |  |  | .50 | .10 |
| Intolerance of Uncertainty (IU) | 1.77 | .11 | .33*** |  |  |
| IU Square Term | .39 | .08 | .09*** |  |  |
| Block 5 |  |  |  | .51 | .01 |
| COVID-19 CGA | -.36 | .07 | -.09*** |  |  |

1. **Discussion of Control Variable Results**

Consistent with existing research, poorer self-rated physical health (Vindegaard & Benros, 2020), greater financial comfort (Isaacs et al., 2018), and greater perceived social support (Van Lente et al., 2012) were associated with better mental health. Extending this research, we found that a more positive impact of COVID-19 on daily life was associated with greater mental well-being as well as perceptions of its recovery. However, negative COVID-19 impact played a relatively weaker role in mental well-being levels and did not predict perceptions of recovery. Consistent with personality research, lower neuroticism was shown to be important predictor for mental well-being, as were higher extraversion and agreeableness (Anglim & Grant, 2016; Grant et al., 2009; Keyes et al., 2002). For the perceptions of mental well-being recovery, extraversion was the most important personality predictor, followed by conscientiousness.

For mental health outcomes, significant predictors were mostly consistent across the two models predicting Kessler-10 and GAD-7 scores. Being younger and female predicted greater psychological distress and anxiety levels. This is consistent with results of the 2017/2018 National Health Survey indicating higher distress and anxiety levels in females and younger age groups (Australian Bureau of Statistics, 2018). Not residing in NSW was a significant predictor for distress, but not anxiety. Replicating exiting findings, having more chronic health conditions and health risk factors were both associated with elevated distress and anxiety (Alonzi et al., 2020). Both positive and negative COVID-19 impact were positive predictors of both outcomes; however, negative impact was markedly stronger, having a greater effect on mental health. One possible explanation for positive impacts being associated with greater distress and anxiety (albeit very weakly) is that higher scores on the impact variables indicate greater change and change itself may elicit distress and anxiety. These findings also extend upon the existing research to suggest perceptions that measures are unnecessary and costly was associated with poorer mental health. Regarding level of worry about COVID-19, concerns about oneself and one’s family being infected and about one’s financial situation predicted elevated levels of both distress and anxiety. Additionally, concerns about the economy and social liberties were associated with lower distress, but unrelated to anxiety. In general, this is consistent with Barzilay et al.’s (2020) finding that a composite score of COVID-related worries was strongly related to higher GAD7 and depression scores. To our knowledge, this is the first study to separately report associations between different dimensions of COVID-19 worries and mental health metrics.

**References**

Abbott, A. (2021). COVID’s Mental-health toll: Scientists track surge in depression. *Nature, 590*, 194-195.

Alonzi, S., La Torre, A., & Silverstein, M. W. (2020). The psychological impact of preexisting mental and physical health conditions during the COVID-19 pandemic. *Psychological trauma: Theory, research, practice and policy*, *12*(S1), S236–S238. https://doi.org/10.1037/tra0000840

American Psychological Association. (2019, October 8). *Manage stress: Strengthen your support network.* https://www.apa.org/monitor/2019/05/ce-corner-isolation

Anglim, J., & Grant, S. (2016). Predicting psychological and subjective well-being from personality: Incremental prediction from 30 facets over the Big 5. *Journal of Happiness Studies*, *17*, 59-80. https://dx.doi.org/10.1007/s10902-014- 9583-7

Australian Bureau of Statistics. (2018, December 12). *Mental health.* <https://www.abs.gov.au/statistics/health/mental-health/mental-health/latest-release>

Australian Bureau of Statistics. (2022a, April 26). *Australia’s Population by Birth Country.* <https://www.abs.gov.au/statistics/people/population/australias-population-country-birth/latest-release>

Australian Bureau of Statistics. (2022b, June 28). *National, state and territory population.* <https://www.abs.gov.au/statistics/people/population/national-state-and-territory-population/latest-release#states-and-territories>

Barzilay, R., Moore, T. M., Greenberg, D. M., DiDomenico, G. E., Brown, L. A., White, L. K., Gur, R. C., & Gur, R. E. (2020). Resilience, COVID-19 related stress, anxiety and depression during the pandemic in a large population enriched for healthcare providers. *Translational Psychiatry, 10*, 291. <https://doi.org/10.1038/s41398-020-00982-4>

Coulombe, S., Pacheco, T., Cox E., Khalil, C., Doucerain, M. M., Auger, E., & Meunier S. (2020). Risk and resilience factors during the COVID-19 pandemic: A snapshot of the experiences of Canadian workers early on in the crisis. *Frontiers in Psychology*, *11,* 3225. https://doi.org/10.3389/fpsyg.2020.580702

Dalgard, O. S. (1996). Community mental health profile as tool for psychiatric prevention. In D. R. Trent & C. Reed (Eds.), *Promotion of mental health.* Aldershot: Avebury.da

Gangopadhyaya, A., & Garrett, A. B. (2020). Unemployment, health insurance, and the COVID-19 recession. *SSRN*. <https://dx.doi.org/10.2139/ssrn.3568489>

Grant, S., Langan-Fox, J., & Anglim, J. (2009). The Big Five traits as predictors of subjective and psychological well-being. *Psychological Reports*, *105*(1), 205-231. <https://doi.org/10.2466%2FPR0.105.1.205-231>

Gubler, D.A., Makowski, L. M., Troche, S. J., & Schlegel, K. (2021). Loneliness and well-being during the covid-19 pandemic: Associations with personality and emotion regulation. *Journal of Happiness Studies, 22,* 2323–2342. <https://doi.org/10.1007/s10902-020-00326-5>

Hampshire, A., Hellyer, P.J., Soreq, E. et al. (2021). Associations between dimensions of behaviour, personality traits, and mental-health during the COVID-19 pandemic in the United Kingdom. *Nature Communications, 12,* 4111. <https://doi.org/10.1038/s41467-021-24365-5>

Isaacs, A. N., Enticott, J., Meadows, G., & Inder, B. (2018). Lower income levels in Australia are strongly associated with elevated psychological distress: Implications for healthcare and other policy areas. Frontiers in Psychiatry, 9, 536. https://doi.org/10.3389/fpsyt.2018.00536

Keyes, C. L., Shmotkin, D., & Ryff, C. D. (2002). Optimizing well-being: The empirical encounter of two traditions. *Journal of Personality and Social Psychology*, *82*(6), 1007-1022.

Steel, P., Schmidt, J., & Shultz, J. (2008). Refining the relationship between personality and subjective well-being. *Psychological Bulletin, 134*(1), 138-161. <https://doi.org/10.1037/0033-2909.134.1.138>

Van Lente, E., Barry. M. M., Molcho, M., Morgan, K., Watson, D., Harrington, J., & McGee, H, (2012). Measuring population mental health and social well-being. *International Journal of Public Health, 57*(2), 421-420. <https://doi.org/10.1007/s00038-011-0317-x>

Vindegaard, N., & Benros, M. E. (2020). COVID-19 pandemic and mental health consequences: Systematic review of the current evidence. *Brain, Behaviour, and Immunity, 89*, 531-542. <https://doi.org/10.1016/j.bbi.2020.05.048>

World Health Organization & Calouste Gulbenkian Foundation. (2014). Social determinants of mental health. <https://www.who.int/mental_health/publications/gulbenkian_paper_social_determinants_of_mental_health/en/>
